# Covalent integration of polymers and porous organic frameworks

**DOI:** 10.3389/fchem.2024.1502401

**Published:** 2024-12-18

**Authors:** Md Amjad Hossain, Kira Coe-Sessions, Joe Ault, Felix O. Gboyero, Michael J. Wenzel, Bhausaheb Dhokale, Alathea E. Davies, Qian Yang, Laura de Sousa Oliveira, Xuesong Li, John O. Hoberg

**Affiliations:** ^1^ Department of Chemistry, University of Wyoming, Laramie, WY, United States; ^2^ Center for Advanced Scientific Instrumentation, University of Wyoming, Laramie, WY, United States

**Keywords:** polymer, porous organic frameworks, metal-organic frameworks, covalent organic frameworks, hydrogen-bonded organic frameworks

## Abstract

Covalent integration of polymers and porous organic frameworks (POFs), including metal-organic frameworks (MOFs), covalent organic frameworks (COFs) and hydrogen-bonded organic frameworks (HOFs), represent a promising strategy for overcoming the existing limitations of traditional porous materials. This integration allows for the combination of the advantages of polymers, i.e., flexibility, processability and chemical versatility etc., and the superiority of POFs, like the structural integrity, tunable porosity and the high surface area, creating a type of hybrid materials. These resulting polymer-POF hybrid materials exhibit enhanced mechanical strength, chemical stability and functional diversity, thus opening up new opportunities for applications across a large variety of fields, such as gas separation, catalysis, biomedical applications, environmental remediation and energy storage. In this review, an overview of synthetic routes and strategies on how to covalently integrate different polymers with various POFs is discussed, especially with a particular focus on methods like polymerization within, on and among POF structures. To investigate the unique properties and functions of these resultant hybrid materials, the characterization techniques, including nuclear magnetic resonance spectroscopy (NMR), Fourier transform infrared spectroscopy (FTIR), X-ray diffraction (XRD), thermogravimetric analysis (TGA), transmission electron microscopy (TEM) and scanning electron microscopy (SEM), gas adsorption analysis (BET) and computational modeling and machine learning, are also presented. The ability of polymer-POFs to manipulate the pore environments at the molecular level affords these materials a wide range of applications, providing a versatile platform for future advancements in material science. Looking forward, to fully realize the potential of these hybrid materials, the authors highlight the scalability, green synthesis methods, and potential for stimuli-responsive polymer-POF materials as critical areas for future research.

## 1 Introduction

Porous organic frameworks (POFs), including metal-organic frameworks (MOFs) (Yaghi et al., 1995; Li et al., 1999; Cohen, 2012; Redfern and Farha, 2019), covalent organic frameworks (COFs) (Côté et al., 2005; Feng et al., 2012; Bisbey and Dichtel, 2017a) and supramolecular organic frameworks (SOFs) (Tian et al., 2016; 2017; Li et al., 2020; Li et al., 2022; Lin and Chen, 2022; Pedrini et al., 2023) (e.g., the well-known example is hydrogen-bonded organic frameworks (HOFs) (Comotti et al., 1999; He et al., 2011; Hisaki et al., 2019; Lin et al., 2019; Li et al., 2020; Song et al., 2022), have gathered considerable attention due to their tunable porosity, high surface area, and diverse functionalities (Cohen, 2012; Feng et al., 2012; O’Keeffe and Yaghi, 2012; Waller et al., 2015; Redfern and Farha, 2019; Zhao et al., 2021; Zhang et al., 2022; Tan et al., 2023). These unique properties permit POFs to become candidates for a wide variety of applications, including gas storage and separation, catalysis, sensing, and drug delivery (Liu R. et al., 2021; Zhao et al., 2021; Chen et al., 2022; Guan J. et al., 2022). Even with these remarkable attributes, however, POFs often display a number of drawbacks, like poor stability, low mechanical strength, and limited chemical diversity. These challenges have continuously motivated researchers to explore a range of tactics for covalently hybridizing POFs with polymers in order to improve their overall performance, structural integrity, and functional variety (Barcus et al., 2022; Nishijima et al., 2022). By covalently integrating polymers with POFs, researchers can leverage the inherent advantages of both materials to overcome existing limitations and achieve synergistic enhancements in properties and functionalities (Bindra et al., 2023; Lee et al., 2023).

Covalent integration of polymers with POFs provides several unique features. First of all, polymers enable POF structures to benefit from mechanical reinforcement, which increases their stability and durability across a range of working environments. Additionally, the numerous chemical functionalities inherent in polymers offer precise control over the chemical properties of POFs’ surface and pore environments, enabling tailored interactions with guest molecules and improved application performance. Moreover, the presence of polymers makes it possible to manipulate the functions of POF by adjusting the physical characteristics of pores and surfaces. Last but not least, polymers’ natural processability and scalability provide opportunities for the facile synthesis and scaling-up of POF hybrid materials, increasing their suitability for applications in industry.

In this review, we explore the recent advances and key developments in the covalent integration of polymers with diverse types of POFs. We discuss the rationale behind it, highlighting different synthetic routes to integrate polymers with POFs. Subsequently, characterizations and distinctive properties of these polymer-POFs hybrids are addressed, providing the unique advantages and opportunities they offer due to their tailored properties and enhanced performance. Afterwards, the exciting applications of polymer-POF hybrids are discussed in diverse fields of research and technology. Lastly, we provide a conclusion on polymer-POFs hybrids and our perspective about the future direction of research and potential applications of this field. Porous aromatic frameworks (PAFs) represent an important class of porous materials, distinguished by porous yet rigid structures built from aromatic building blocks (Ben et al., 2009; Yuan and Zhu, 2019). They are not discussed separately in this review, as recent reviews have comprehensively covered their synthesis, characterization, and properties (Ben and Qiu, 2013; Tian and Zhu, 2020; Tian et al., 2024). Readers are referred to these reviews for further information on PAFs.

## 2 Synthetic routes of covalent integration of polymer-POFs

Given that POFs are solid materials involving subnano-/nano-sized pores and polymers are in general flexible, we categorize the hybrids into three groups, namely 1) polymers within POFs, 2) polymers onto the surfaces of POFs and 3) polymers among POFs particles. Following this categorization, as below, we review in detail each category of the synthesis of these polymers-POF hybrid materials with specified explanations and illustrative references.

### 2.1 Polymers within POFs

Thanks to the porous nature of POFs, a variety of polymers can be synthesized within their pores using several approaches. As the pore size of POFs is large enough, it can encapsulate an ample number of monomers, allowing polymerization to occur within the pores, resulting in polymers that are distinct from the POF structures themselves. When the ligands of POFs contain polymerizable functional groups, these functional groups can undergo polymerization directly, crosslinking the ligands of POFs and yielding covalently-conjoined polymer networks. Another approach to synthesize the polymer-POFs hybrids involves using POFs with ligands that bear polymerization initiators. In the presence of monomer of polymer, these ligands can initiate polymerization, resulting in the synthesis of polymer with a variety of topology. Additionally, if a POF ligand contains more than one polymerizable group, copolymerization with other monomers can occur, leading to the formation of copolymers.

#### 2.1.1 POFs as template for polymer synthesis

The porous structures of POFs can serve as excellent templates to facilitate the polymerization of a wide variety of monomers within their pores. This approach allows for the synthesis of polymer with unique morphologies and tailored properties that are directly influenced by the confined environments within POFs. By utilizing the well-defined and tunable pore sizes of POFs, it also enables precise control over polymer growth and topology, providing a valuable approach for the design and synthesis of advanced materials. Several notable studies exemplify this strategy, demonstrating innovative techniques and applications of polymerization within POF pores, which are highlighted in the following discussion.

**FIGURE 1:**
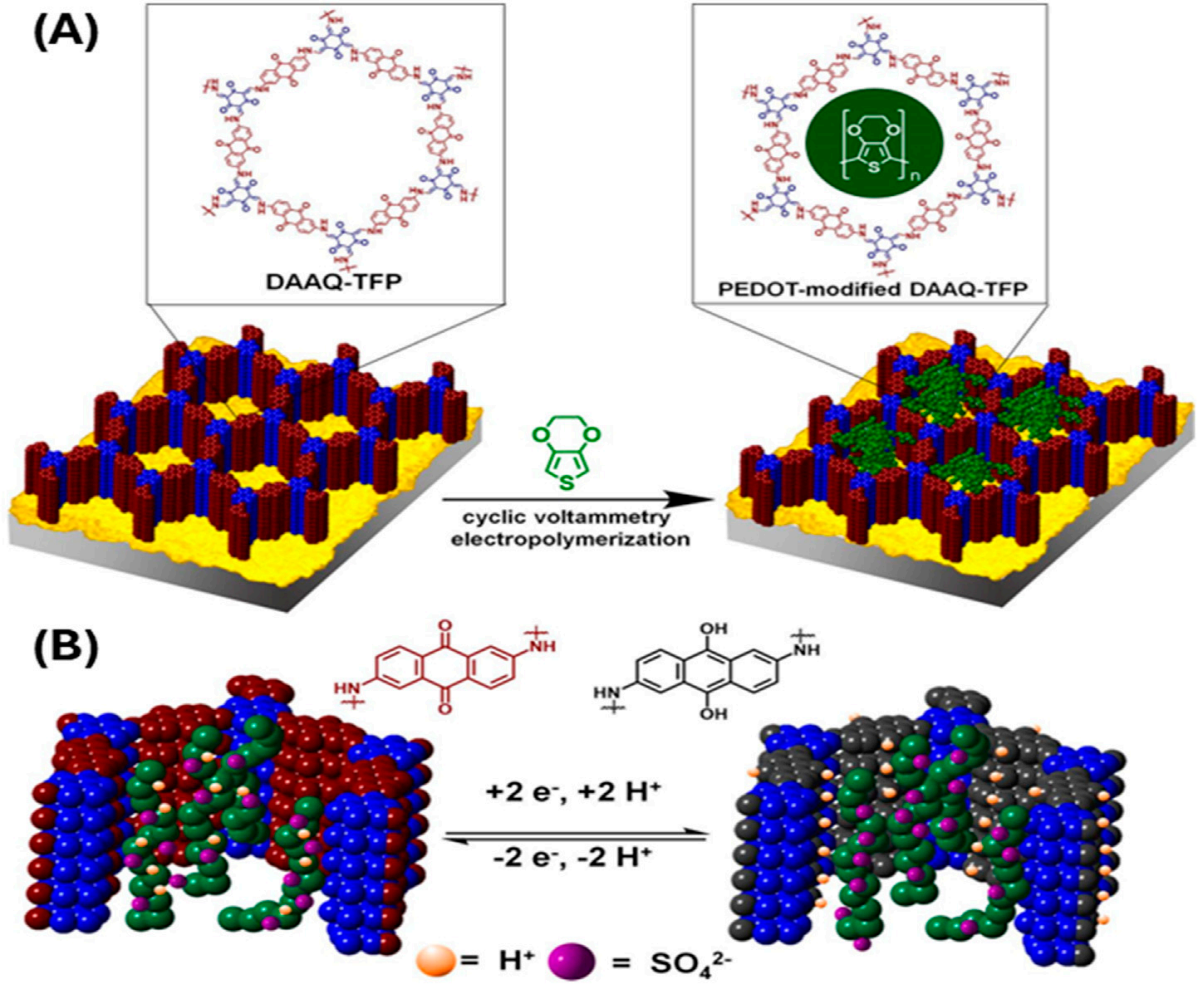
**(A)** Electropolymerization of PEDOT inside the COF DAAQ-TFP. **(B)** Cross-section of poly (3,4-ethylenedioxythiophene) in (PEDOT)-infiltrated COF (PEDOT-DAAQ-TFP) highlighting the electrochemistry of redox active DAAQ moiety. The incorporation of PEDOT makes the pores deep within the flakes accessible electrochemically. [Adapted with permission from ref (Mulzer et al., 2016)].

Dichtel’s group (Mulzer et al., 2016) introduced 3,4-ethylenedioxythiophene (EDOT) monomer in a redox active COF and electropolymerized it within the COF frameworks resulting poly (3,4-ethylenedioxythiophene) in (PEDOT)-infiltrated COF (PEDOT-DAAQ-TFP) (Figure 1). The resulting polymer within COF (PEDOT-DAAQ-TFP) significantly enhances the electrical conductivity and electrochemical response. This enables the quantitative access to the redox active groups even at 1 µm thick flakes. These materials show superior charging rates (of 10–1600 C) and 10-fold superior current response with stable capacitances for 10,000 cycles.

**FIGURE 2:**
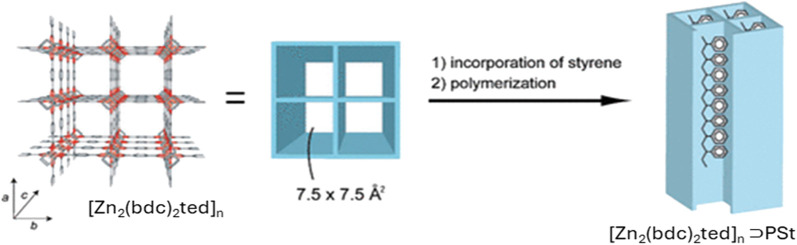
Nanocomposite depicting the confinement of single polystyrene (PSt) chain within the MOF with [Zn_2_ (bdc)_2_ted]_*n*_ [Adapted with permission from ref (Uemura et al., 2008)].

Kitagawa’s group (Uemura et al., 2008) synthesized a nanocomposite of Polystyrene (PS) with [Zn_2_ (bdc)_2_ted]_*n*_⊃PSt (bdc = 1,4-benzenedicarboxylate, ted = triethylenediamine) by radical polymerization of adsorbed styrene inside the nano channels of MOFs (Figure 2). The resulting PS shows different properties than the bulk PS as the confinement inside the MOF promotes homogeneous side-chain mobility and low activation energy. PS obtained here does not show any glass transition in colorimetric analysis contrary to its bulk analogue and the PS chains in MOF show linear extension of chains and lacking chain-chain interactions.

Additionally, Uemaro’s group performed the polymerization of thiophene inside nanoporous coordination template (P [La(BTB)]_n_ (BTB = 1,3,5-benzentrisbenzoate) (Kitao et al., 2017). The synthesized polymer showed extended conjugation and superior conductivity than that of polythiophene prepared by solution polymerization.

#### 2.1.2 Polymerization of ligands

This method utilizes pre-designed/pre-functionalized ligands containing polymerizable groups. These groups then undergo polymerization reactions, such as condensation or step-growth polymerization, to form a polymer backbone within the POF structure. This approach offers precise control over the polymer’s chemical composition and its distribution within the framework. The outcome of this approach depends on the number of polymerizable groups on each ligand. With a single group per ligand, a homopolymer chain is formed throughout the framework. However, if multiple groups are present, a cross-linked network of polymer chains can be achieved, further influencing the pore structure and stability of the POF. To illustrate this method, some helpful examples are presented as follows.

**FIGURE 3:**
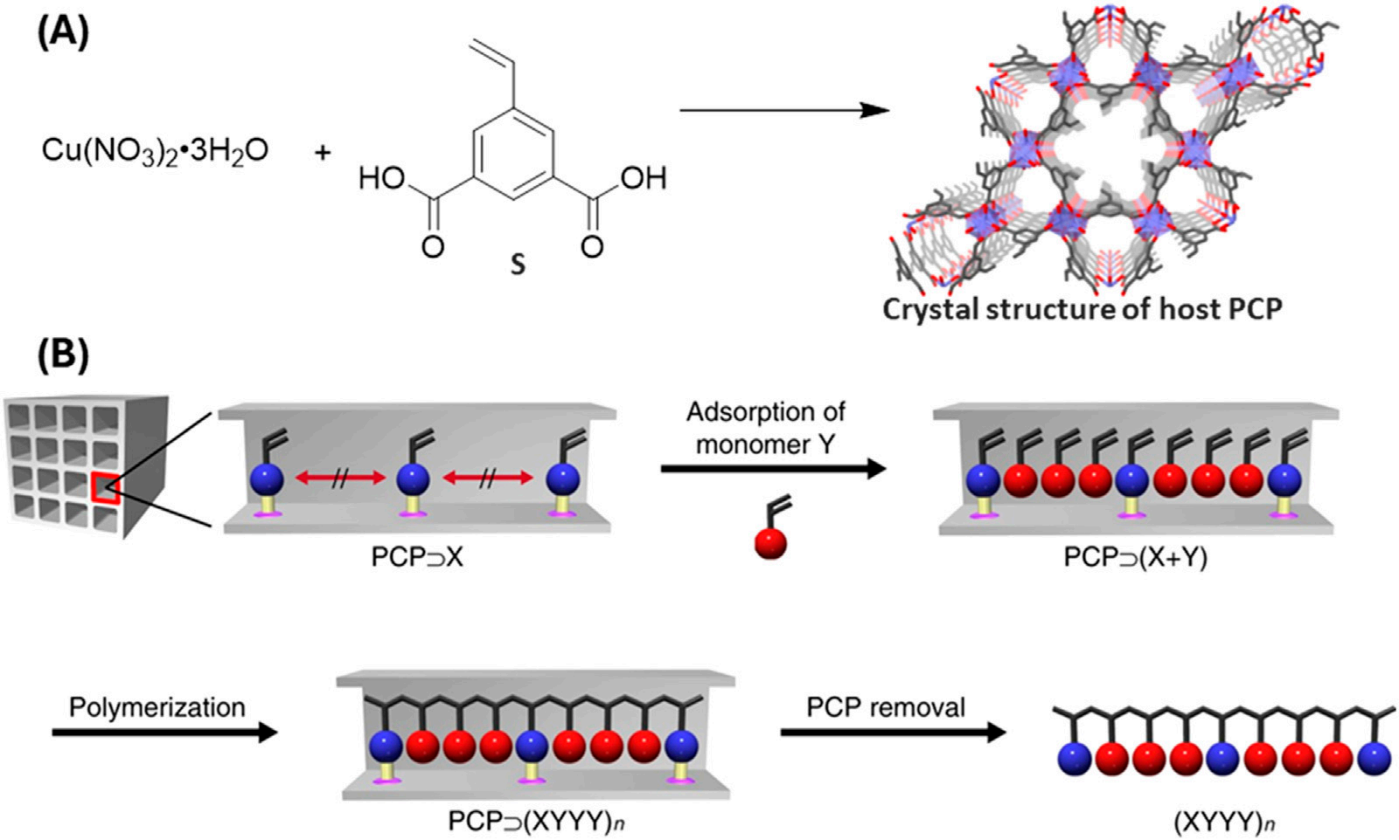
**(A)** Synthesis and crystal structure of PCP. **(B)** To create sequence specific co-polymers, a vinyl monomer (X) is first anchored at consistent intervals within the nanochannels of PCP. Following this, a second vinyl monomer (Y) is introduced into the PCP framework, forming a host–guest composite (PCP⊃(X + Y)). The monomers undergo polymerization by AIBN initiator within the composite. The removal of PCP host by treatment with 1M HCl, yields sequence regulated copolymer. [Reproduced with permission from ref (Mochizuki et al., 2018)].

Uemura’s group (Mochizuki et al., 2018) employed a strategy of controlled polymerization inside a MOF pore and of MOF ligand for the synthesis of sequence specific co-polymer (Figure 3). MOF’s periodic structure has been utilized as a template to control monomer sequence. Styrene-3,5-dicarboxylic acid (S) was used to create a porous coordination polymer (PCP), [Cu(styrene-3,5-dicarboxylate)]_n_ with styryl groups positioned along channels. Acrylonitrile (A) was then introduced and copolymerized with the styrene (S). The resulting copolymer has a SAAA sequence, which successfully provides a novel method to synthesize sequence-controlled polymer thanks to the precise structure of POFs.

**FIGURE 4:**
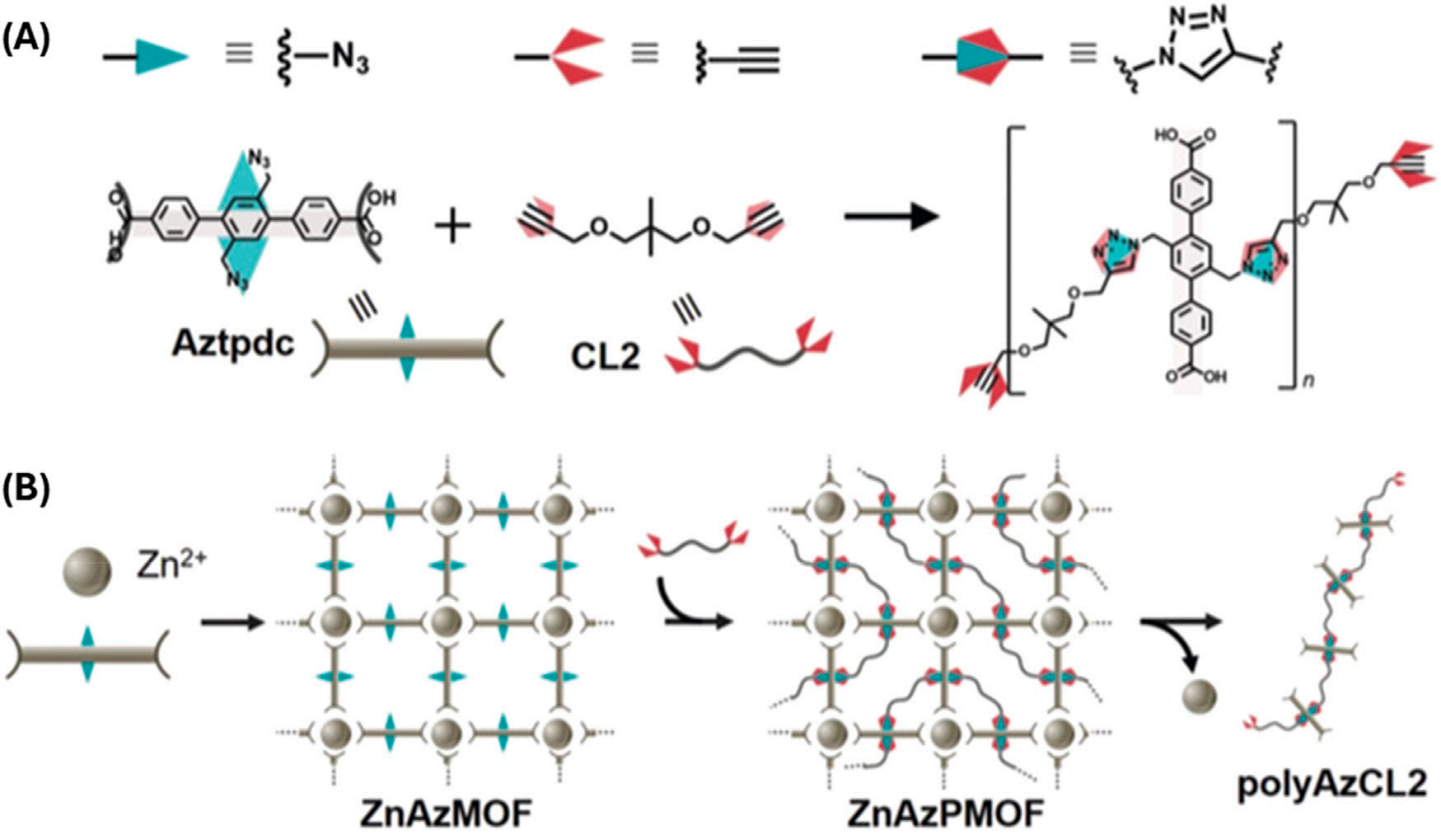
**(A)** The azide–alkyne cycloaddition polymerization of azide, Aztpdc and alkyne, CL2 to form triazole **(B)** Copolymerization of two ligands in different environments within MOF. One ligand is immobilized within MOF framework while the another is a mobile inside the pores of MOF. [Adapted with permission from ref (Anan et al., 2019)].

Sada and Kokado used organic ligands within MOFs to create polymers, (Anan et al., 2019), by immobilizing one monomer within the MOF while another mobile monomer polymerized through the pores of the MOF (Figure 4). The POF (MOF) was synthesized using Zn(II) and ligand containing two azide groups. In the next step, the dual alkyne functionalized monomer was reacted with Cu(I) catalyst for click reaction. This unique approach leads to polymerizations with specific degrees of polymerization. This method provides a promising means to control the step-growth copolymerization.

#### 2.1.3 Polymerization from ligands

This strategy leverages multifunctional ligands that act as both building blocks and initiators for polymerization. These ligands possess functionalities that can initiate the polymerization of additional monomers, leading to the formation of polymers directly within the POF framework. The nature of the incorporated monomers dictates the resulting polymer type, allowing for the creation of homopolymers, random copolymers, alternating copolymers, block copolymers, and even graft copolymers.

**FIGURE 5:**
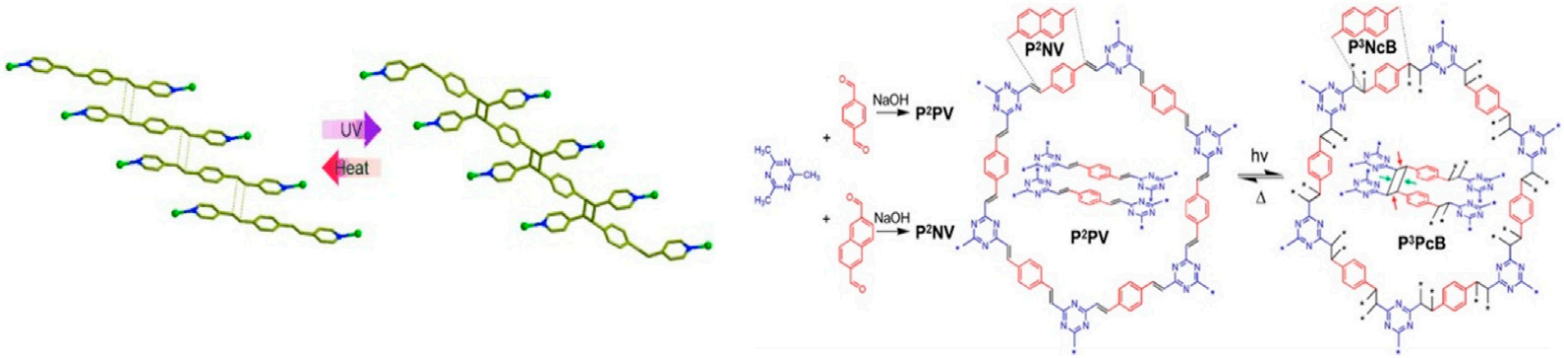
Reversible [2 + 2] cycloaddition photo-polymerization of ligands in 2D MOF (left) and COF (right) leading to 3D polymers. [Adapted with permission from ref (Park et al., 2014; Jadhav et al., 2020)].

Additionally, existing polymers can be employed in this approach such that the ligands are functionalized with these pre-synthesized polymers, resulting in polymer-POFs hybrids with tailored properties. The Vitthal and Lee groups (Park et al., 2014; 2015; Medishetty et al., 2016) utilized the double bonded ligands on MOFs for the [2 + 2] cycloaddition photo-polymerization (Figure 5). This polymerization inside the MOF crystal was a single crystal to single crystal polymerization transformation and depends on the alignment of ligands. The photochemical polymerization can be reversed by heat. A similar strategy was used by the Perepichka group (Jadhav et al., 2020) to make 3D COFs from 2D COFs by [2 + 2] photo-cycloaddition of vinyl functionality on COF ligands. The reaction was reversible by heating at 200°C and as such retained the crystallinity of the 2D-COFs.

**FIGURE 6:**
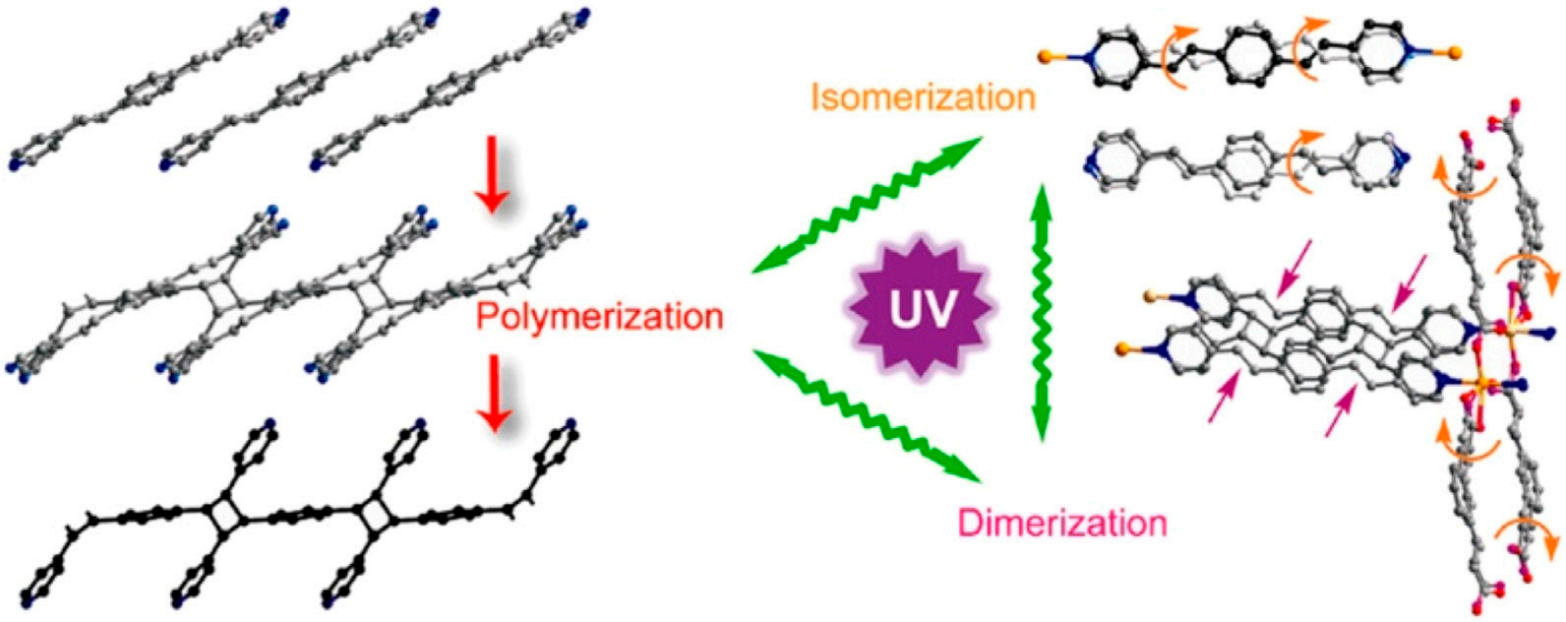
Photopolymerization of photoreactive guests inside the photoreactive POF as a molecular reactor, leading to various photoreactions. [Adapted with permission from ref (Yang et al., 2014)].

Yang and Naumov (Yang et al., 2014) introduced photoactive guest 1,4-bis [2-(4-pyridyl)ethenyl]benzene (1,4-bpeb) and host molecules in channels of photoactive porous coordination polymer composed of 1,3-phenylenediacrylic acid (1,3-pda) with Mn(II) and photopolymerized *via* [2 + 2] cycloaddition (Figure 6). The 1,4-bpeb acts as both ligand and guest and both the ligands (building blocks of the MOF) and guest molecules (introduced into the MOF) can react under light irradiation. This dual reactivity enables light to trigger the formation of polymer chains from a specific molecule (1,4-bpeb) within a solid MOF structure. Importantly, the MOF structure remained intact throughout the polymerization process, even though it became significantly distorted. This highlights the material’s stability during light-driven transformations. The processed photo-polymerization, dimerization and peddle like isomerization, were monitored by single crystal-x-ray crystallography.

#### 2.1.4 Polymerizations between ligands

This method involves exploiting the inherent functionalities of the ligands for covalent integration. Here, two scenarios can occur.

1. **All ligands with polymerizable groups:** When every ligand in the POF structure possesses polymerizable functionalities, the addition of suitable monomers can trigger polymerization, resulting in a network of polymers distributed throughout the framework.
2. **Specific ligand functionalization:** If only a portion of the ligands contain polymerizable groups, polymerization can still occur. However, the resulting polymer distribution and topology will differ from the scenario described above.

**FIGURE 7:**
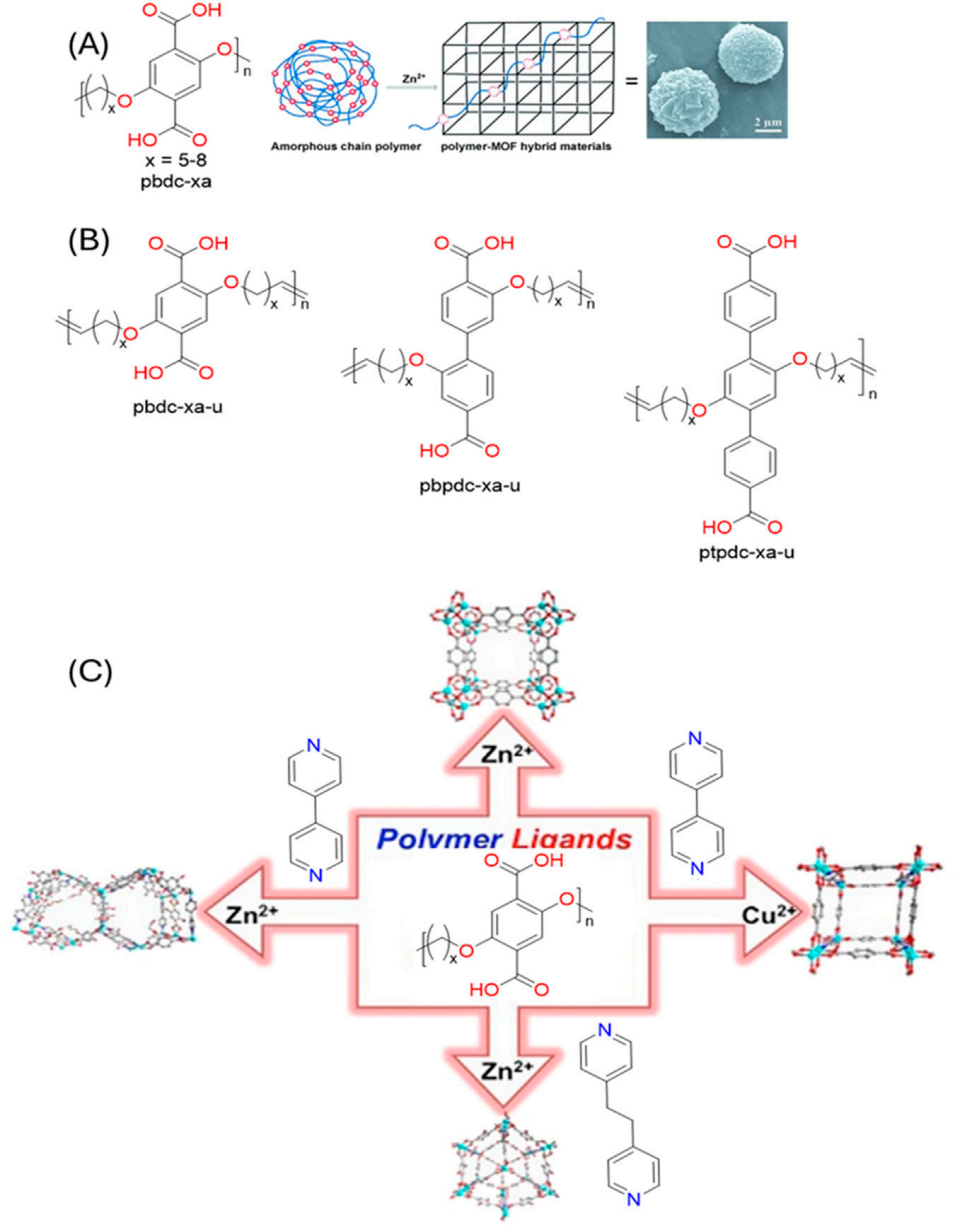
**(A)** Functionalization of amorphous polyether polymers containing aromatic di-carboxylic acid functional groups leading to the crystalline MOFs. **(B)** The variety of polyether ligands utilized. **(C)** Extension of strategy with various metals and co-ligands. [Adapted with permission from ref (Cohen, 2012; Zhang et al., 2016; Schukraft et al., 2017)].

Additionally, if the ligands possess reactive groups, existing polymers can be functionalized within the POFs, leading to the formation of hybrid materials. Cohen’s group (Zhang et al., 2015a) utilized the polyether containing benzene-di-carboxylic acid in backbone and reacted with Zn(II) (Figure 7). The acid groups from the amorphous polymer act as a ligand and form crystalline MOFs with Zn(II). The obtained polymer in MOF was polycrystalline with intergrowth of various crystallites with better hydrophobicity and permanent porosity. The choice of ligands and specific annealing temperatures can be used to get specific morphologies ranging from spherical superstructures to crystalline films. Further the same group extended this strategy to a mixed ligand strategy in presence of another co-ligand (Zhang et al., 2016). Herein they used both polymer ligand and another ligand with a pyridine group. They also extended the strategy to another metal C(II) indicating the generality. The produced polyMOF has increased hydrophobicity and can be used for potential separation of CO_2_ from N_2._ Further Cohen group introduced isoreticular expansion by varying the length of ligands in UiO-66 polyMOF (Schukraft et al., 2017). Use of ligands with phenyl (pbdc-xa-u), biphenyl (pbpdc-xa-u) and terphenyl (ptpdc-xa-u)backbone provided larger surface areas and higher stability. The polyMOFs exhibit greater flexibility than their MOF counterparts making them more resistant to mechanical stress and improving the processibility. The polyMOFs are more porous and ordered than polymers. Like MOFs their properties are tunable based on the nature of metal node and organic linkers.

### 2.2 Polymers on POFs

**FIGURE 8:**
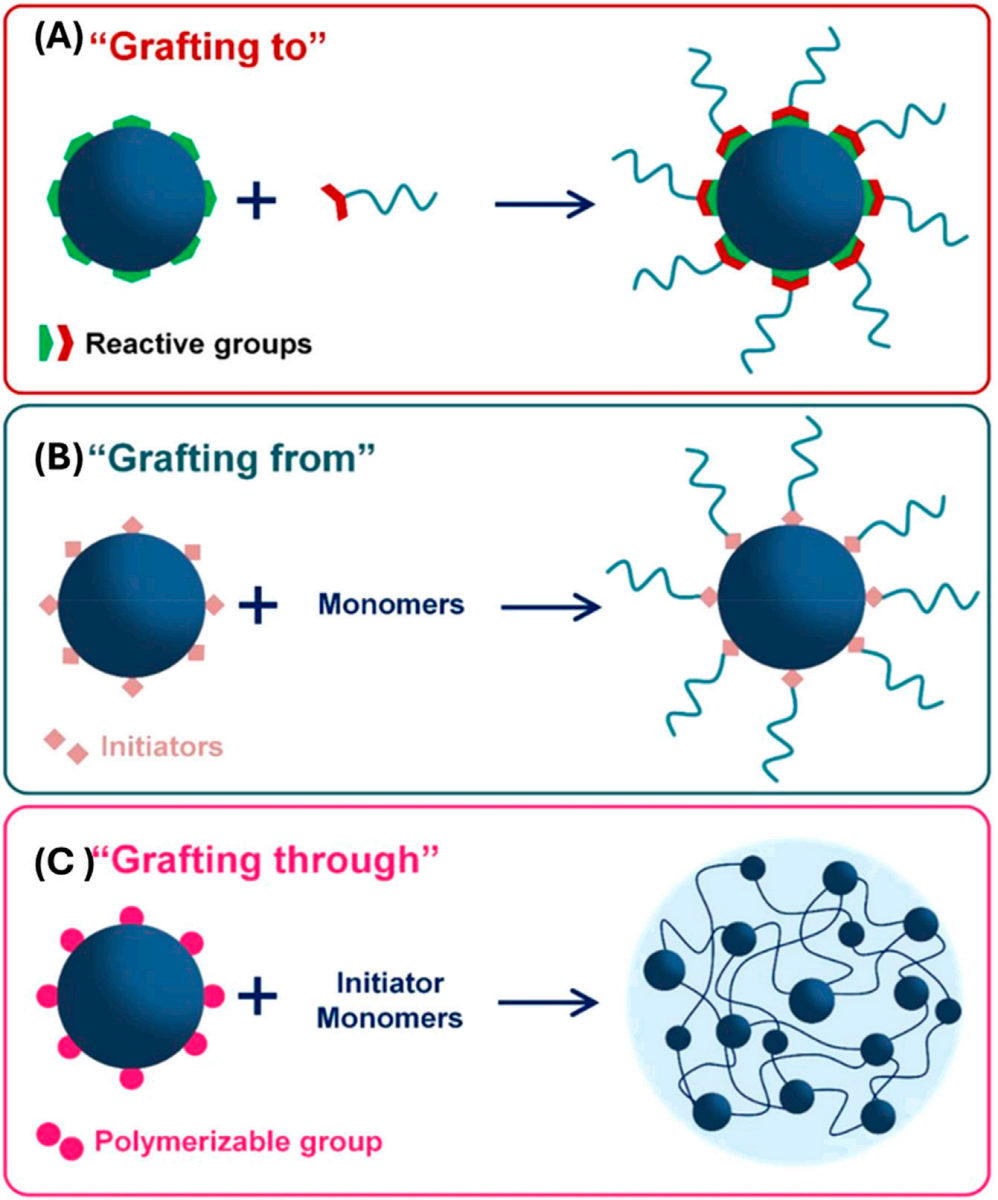
“Strategies of polymer grafting: **(A)** grafting to, **(B)** grafting from and **(C)** grafting through.” Published with permission (Macchione et al., 2018).

The formation of polymers on the surface of POFs is an emerging area of research that offers unique opportunities for synthesizing novel polymer structures and can also expand the knowledge on polymerization mechanisms at the molecular level through advanced instrumentation. POFs, such as COFs, MOFs, and SOFs, are known for their crystalline nature, versatile design, and functional pore. However, with the addition of a polymer the resulting hybrid material can achieve enhanced properties with increased conductivity (Yang S. et al., 2021), thermal stability (Shen et al., 2015), and overall performance (Efome et al., 2018) of the new hybrid composition. There are three primary polymerization formation categories that can be identified: “graft-to,” “graft-from,” and “graft-with,” as illustrated in Figure 8. “Graft-to” describes a polymer physically connected to the surface of a POF typically through covalent bonds. In this situation the polymer has been pre-synthesized and is just chemically attached to the POF particle. The term “graft-from”, simply put, describes on-surface polymerization, which links organic building blocks directly to a two-dimensional surface (Nacci et al., 2016). The polymer grows *in situ* and relies on intermolecular forces to keep the polymer on the POF. Lastly, “graft-with” is a recent idea in which the polymer and the POF are formed simultaneously.

#### 2.2.1 Graft-from

**FIGURE 9:**
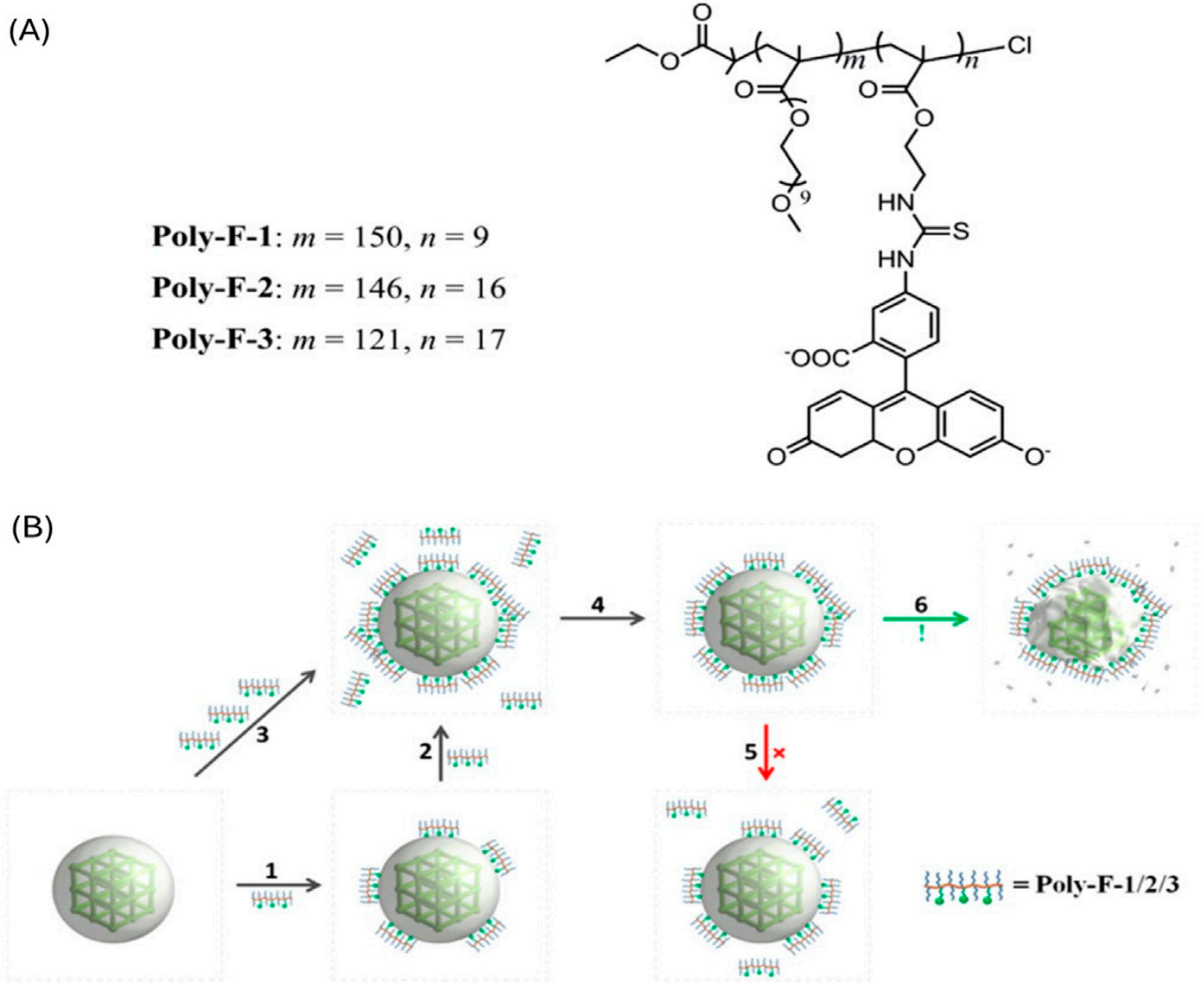
**(A)** Structure of pOEGMA/pAEMA copolymer–fluorescein conjugates and **(B)** diagram illustrating the binding/assembly of polymers onto the surface of MIL-101-NH_2_ (Fe). Reprinted with permission (Liu et al., 2014).

The most detrimental factor of on-surface polymerization is the selective choice of a monomer. This specific monomer requires site-selective reactivity to form the polymer, while also being stable under the reaction conditions. Most of these monomers are self-reactive and create a homopolymer. Therefore, the polymer and POF need to have intermolecular interactions such as hydrogen bonding (He et al., 2019), π-π stacking (Skorupskii et al., 2022), or electrostatic interactions (Liu et al., 2014) to ensure the polymer is truly on the surface of the POF. Liu et al. systematically studied the noncovalent interactions of an iron (III) carboxylate nano-MOF, MIL-101-NH_2_ (Fe), with molecular probes containing negative charges and hydrophobic properties. As illustrated in Figure 9, a polymer with fluorescein side chains can utilize cooperative binding to attach to the surface of the MOF in aqueous conditions, enabling fluorescent imaging and drug delivery. The addition of the polymer on the MOF nanoparticles surface resulted in the nanoparticles stability increasing and therefore decreased the degradation of the MOF nanoparticles in an aqueous environment. Unlike most polymer coated MOFs this work shows that the polymers are non-exchangeable and are notably “nonsheddable” in water (Liu et al., 2014).

Nuemann et al. took a different approach by threading a liquid crystalline polymer through a COF. These crystalline polymers entangled in the COF enhance the mechanical properties by enabling the energy from a fracture to disperse evenly throughout both the COF and the polymer. Two different polymers, polymethyl methacrylate (PMMA) and polyimide (PI), were used to determine if an amorphous, brittle polymer would interact similarly to a crystalline polymer that closely resembles the COF backbone. In both cases the microbridges formed and helped distribute tension energy as ascertained by tensile testing. However, after extensive characterization it was shown that the PMMA polymer formed more on-surface interactions whereas the PI created abundantly more molecular woven nanocrystals. These ultimately created an improved nanofibril network and provided additional mechanical strength as well as enhanced filler distribution (Neumann et al., 2024).

**FIGURE 10:**
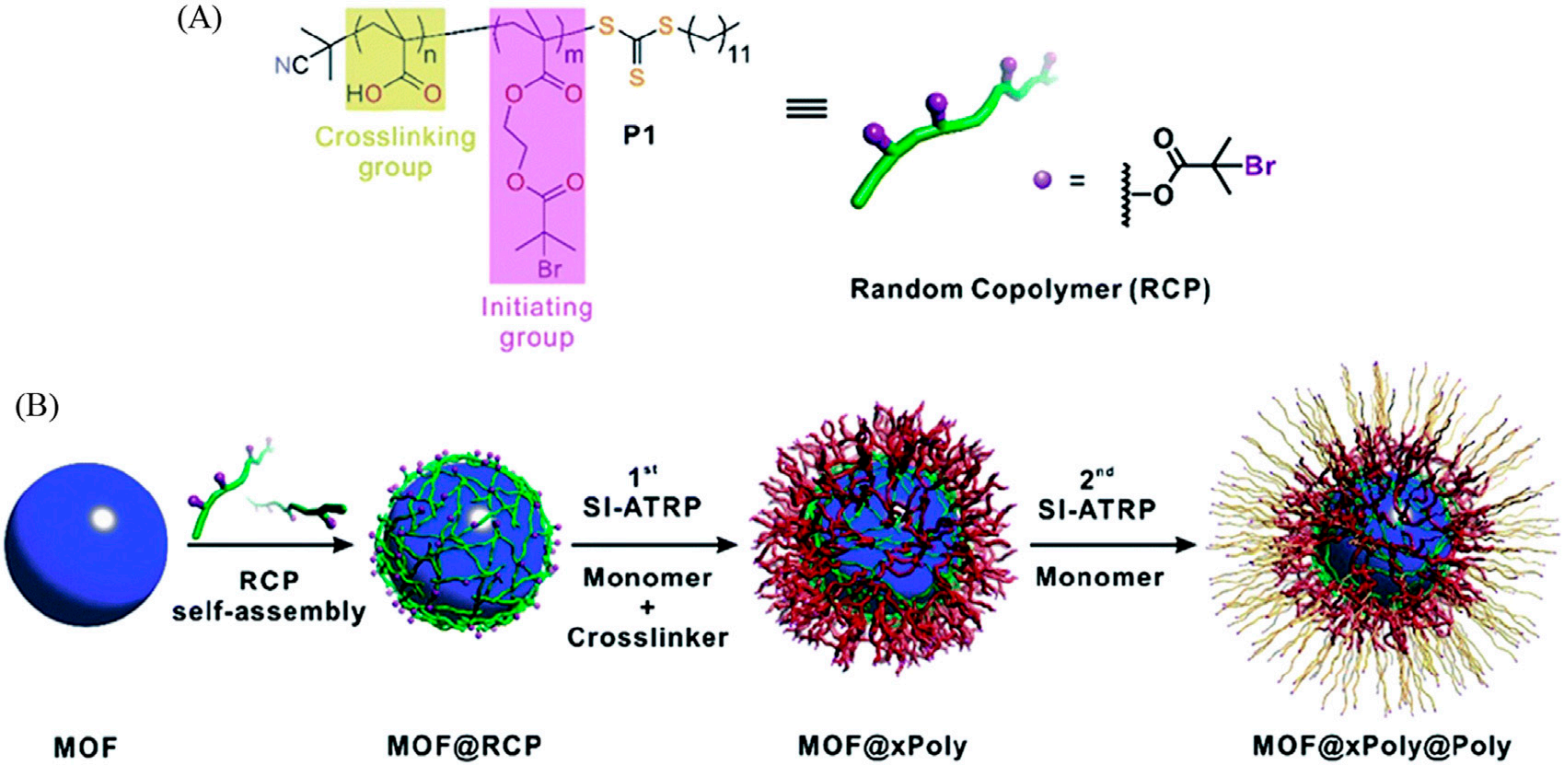
“**(A)** Molecular structure of the RCP macroinitiator, P1. **(B)** Schematic illustration of typical experimental procedures for growing polymer shells on a MOF particle.” Printed with permission (He et al., 2019).

He et al. used a random copolymer (RCP) as a macroinitiator that assembles on the MOF surface through inter-chain hydrogen bond crosslinking, as illustrated in Figure 10. These RCPs show a substantial advantage compared to normal surface-initiated atom transfer radical polymerization (SI-ATRP) monomers. Firstly, they are hydrogen bonded and therefore do not attach to the inside of the pore like typical covalent SI-ATRP monomers. Secondly, they self-assembled separately from the MOF and therefore are adaptable to a variety of MOFs. Lastly, the polymer is covalently cross-linked to the RCP and therefore is very stable and can withstand extreme environments while protecting the integrity and crystallinity of the MOF. Simply stated, the MOF crystal is protected by a hydrogen bonded “cloud” of RCP that has a polymer covalently linked to it. With the additional layer of polymers on the surface of the MOF the porosity surprisingly has not changed and yet the wettability, thickness and functionality can be tuned depending on which MOF and which monomer is used. The added polymer coat has also been proven to protect MOF’s crystallinity from acid and base environments. This combined system could find applications in various fields, such as gas separation, sensing, or controlled release of molecules. The precise design and choice of materials would depend on the desired functionality and the specific requirements of the intended application. Overall, it's a fascinating concept with significant potential for advancing the capabilities of POFs in practical applications (He et al., 2019).

**FIGURE 11:**
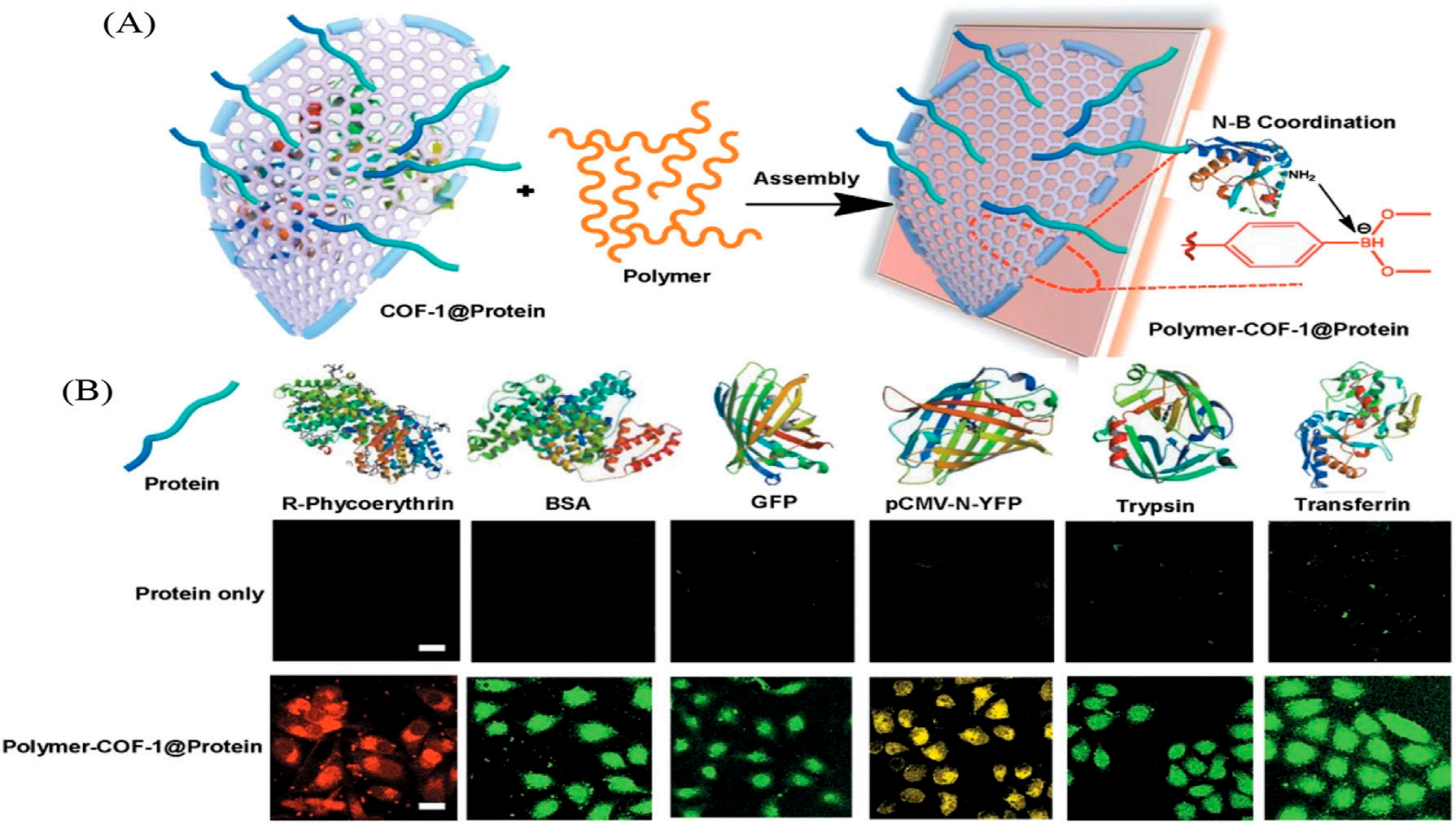
“Boron-rich polymer–COF composites with high efficiency in cytosolic protein delivery. **(A)** Mechanism of boron-rich COF in complexation with protein. The COF could bind with both negatively and positively charged proteins via a combination of nitrogen–boronate complexation interactions between the two species. **(B)** Boron-rich polymer–COF composites show consistent behaviors in the delivery of proteins into A549 cells. Fluorescently labeled BSA was used as the model protein. The doses of protein and polymer–COF (PEG-COF-1@Protein) in each well were 6 and 8 μg, respectively. For all images: scale bar: 25 μm” Reprinted with permission (Zhang et al., 2020).

Zhang et al. created a water-dispersible polymer–COFs nanocomposites (FITC-PEG-COF@Ins-GOx) that uses self-assembly for *in vitro* and *in vivo* insulin delivery as shown in Figure 11. The insulin and glucose oxidase bind to the Boronate COF backbone through Brønsted and Lewis type interactions. The new polymer-COF hybrid material can selectively recognize glucose and release the entrapped insulin by sensing the *in situ* generated hydrogen peroxide and acid environment from the gluconic acid. The fluorescent studies track insulin uptake and release with variations of protein into A549 human cell cultures. Additionally, a mouse model was utilized to assess insulin-delivery nanocarriers aimed at achieving rapid response in type 1 diabetes treatment. This research paves the way for synthesizing durable and effective polymer-COF nanocomposites tailored for cytosolic protein delivery (Zhang et al., 2020).

#### 2.2.2 Graft-to

**FIGURE 12:**
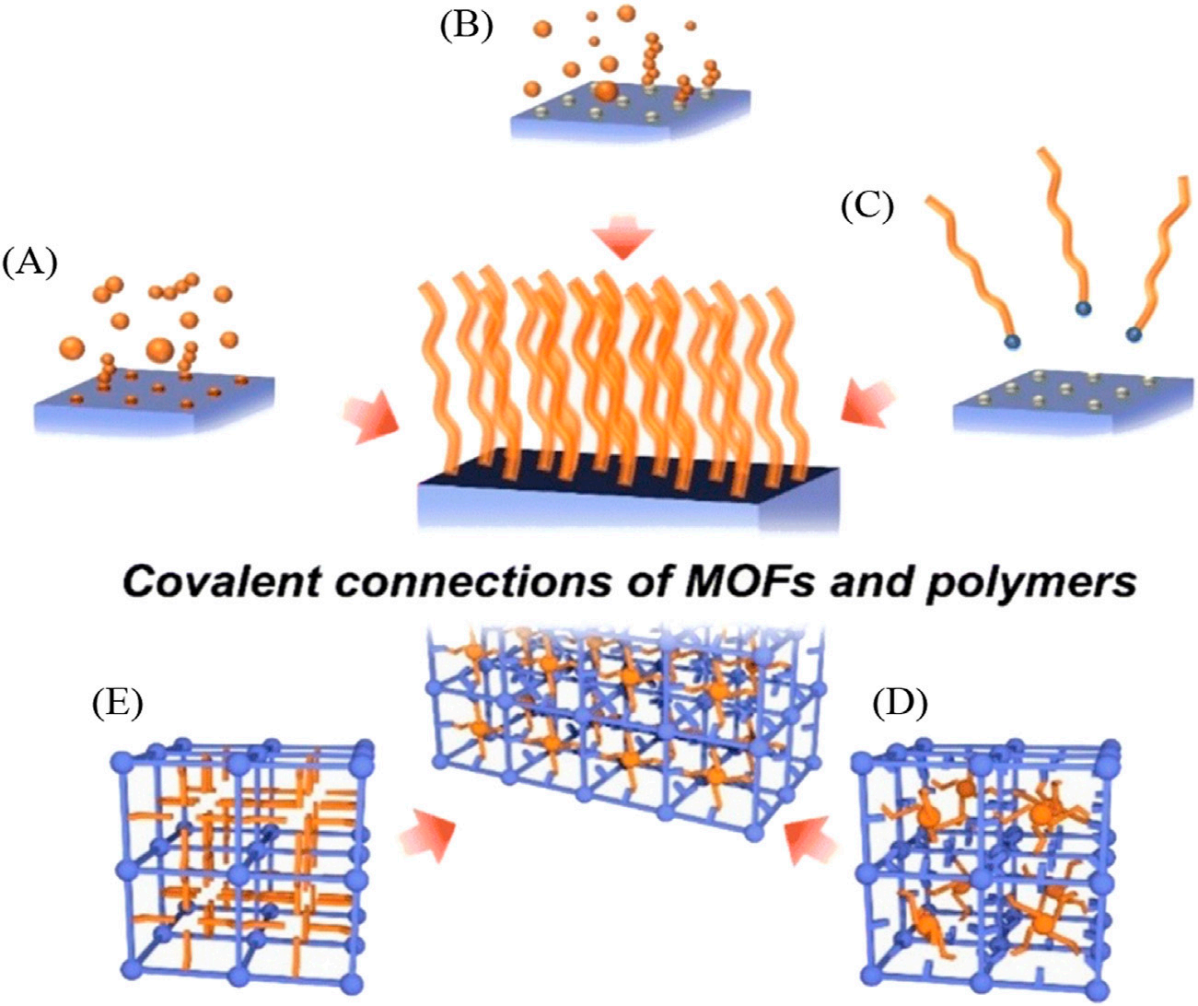
“Schematic illustration of a variety of methods for the covalent hybridization of MOFs and polymers. Grafting polymer to MOFs via **(A)** grafting-through, **(B)** grafting-from, and **(C)** grafting-to approaches; polymerization of frameworks **(D)** with guest molecules and **(E)** without guest molecules.” Reprinted with permission from (Lee et al., 2023).

In the “graft-to” approach, the polymer is physically attached to the MOF surface typically through covalent bonds. This method offers precise control over the attachment of the polymer chains onto the POF surface, potentially leading to a well-defined composite material with tailored properties (Nacci et al., 2016). Figure 12 illustrates various coordination methods by which a polymer can covalently attach to MOFs or ideally to all types of POFs. “Grafting-through” describes a polymer covalently linked in the middle of the POF and shows a form of interweaving coordination. The “grafting-from” method involves initiating the growth of a polymer chain from specific sites on the POF. This results in the formation of a polymer that is covalently bonded to the POF through the initiation sites. The “graft-to” technique involves preformed polymer chains attaching to specific sites on the POF through a covalent reaction. Unlike the “grafting-from” method, the polymer chains are synthesized independently before being attached to the POF.

**FIGURE 13:**
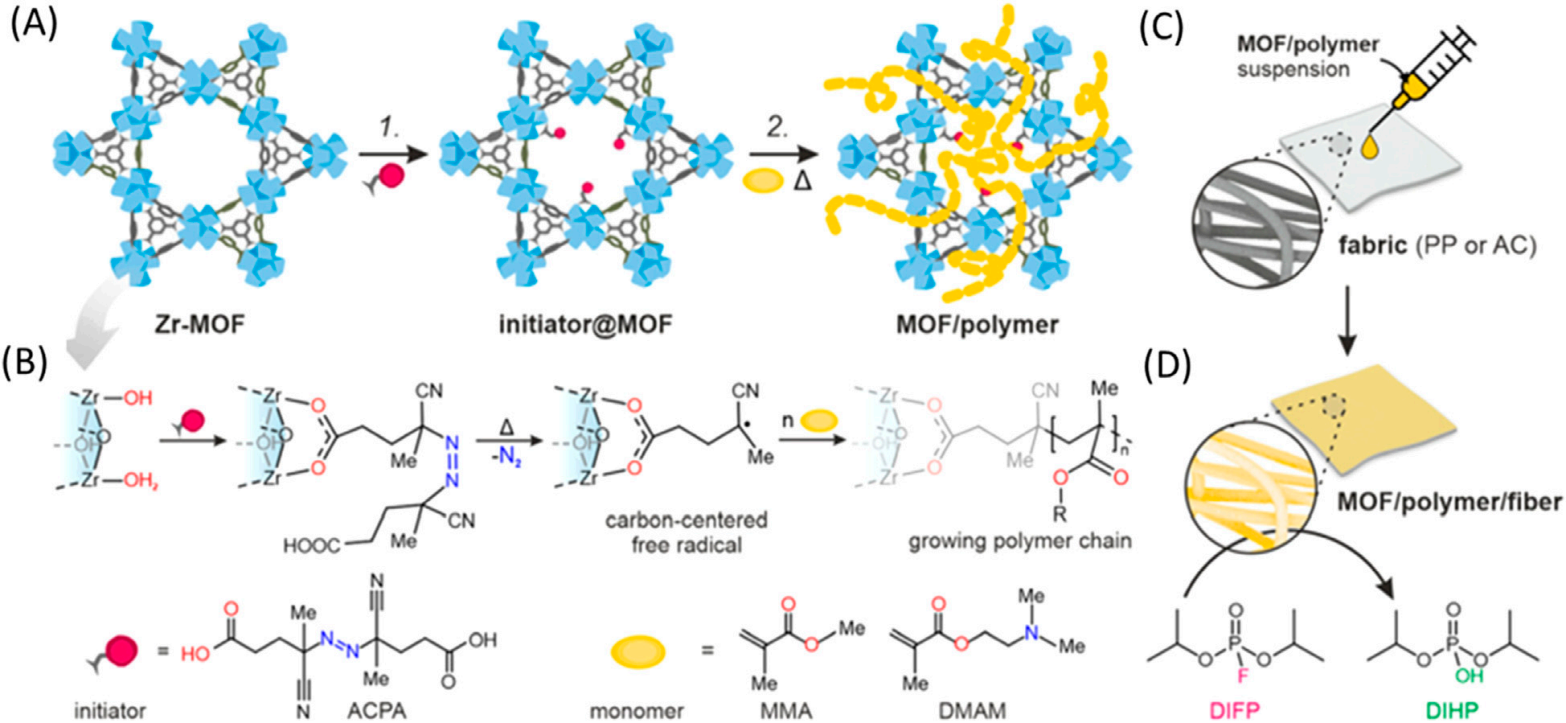
“The two-step protocol for synthesizing MOF/polymer hybrids and their composites with fibers. **(A)** Reaction scheme. Step 1: Solvent-assisted ligand incorporation (SALI) of ACPA (initiator) into a Zr-MOF; step 2: free-radical polymerization in a MOF (FRaP-in-MOF) of acrylate monomers, **(B)** SALI of 6- (MOF-808) or 8-connected (NU-1000) Zr-nodes with ACPA followed by temperature-induced generation of carbon-centered radicals, **(C)** drop-casting of MOF/polymer hybrids on PP and AC fibers, **(D)** hydrolysis of a nerve agent simulant, diisopropyl fluorophosphate (DIFP), catalyzed by the MOF/polymer/fiber composite (conditions: RT, 24 h).” Reprinted with permission (Pander et al., 2023).

Pander et al. (2023) used a Zr-MOF with an azo-initiator to initiate an *in situ* free radical polymerization of an acrylate monomer to create a MOF/polymer hybrid. This new hybrid composite material enhances the adhesion to fibers with the end goal of creating protective clothing capable of detoxifying organophosphorus warfare agents (Figure 13).

**FIGURE 14:**
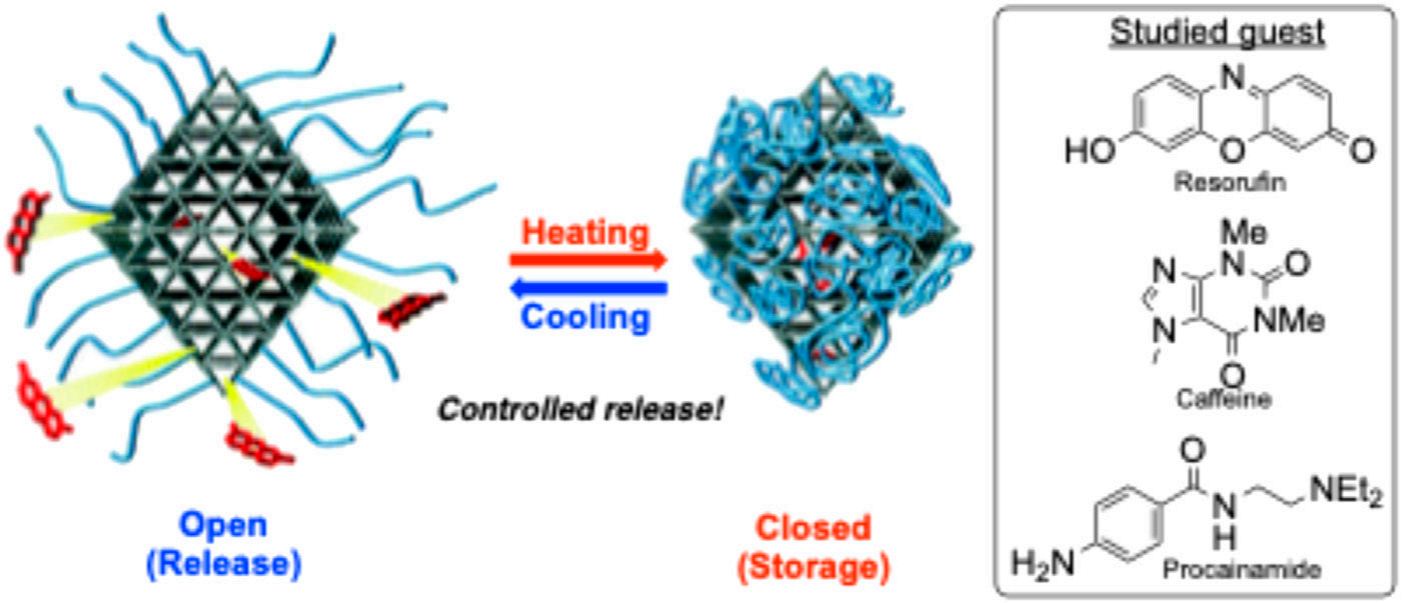
Illustration showing the thermosensitive release of guest polymers through a polymer/MOF hybrid material. Published with permission (Nagata et al., 2015).

A polymer/MOF mixture synthesized by Nagata et al. (2015) used poly (*N*-isopropylacrylamide) (PNIPAM) as a thermosensitive smart polymer to create a precisely controlled “ON-OFF” release (Figure 14). By attaching this polymer post-synthetically onto a Zr(iv) and terephthalate MOF, this new hybrid material can rapidly release certain guest molecules in a specific temperature range. One of the guest molecules tested was caffeine, aligning with the research objective of drug delivery.

**FIGURE 15:**
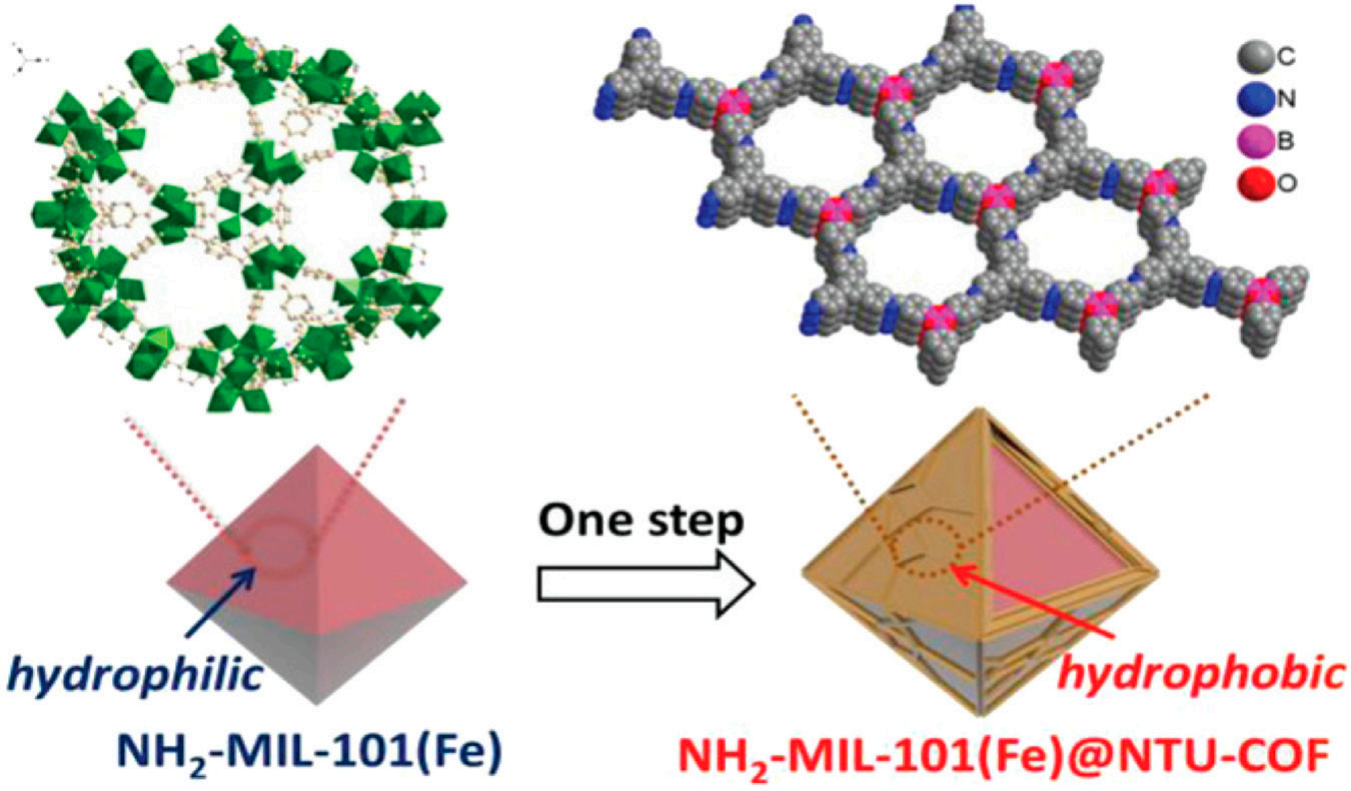
“Fabrication of NH_2_-MIL-101(Fe)@NTU-COF.” Demonstration of the MOF core and an outside COF shell. Reprinted with permission (Cai et al., 2019).

A notable example of “graft-to” would be to create a shell, where the inside core would be a MOF and the outside of the shell would be a COF covalently linked to the core as Cai et al. (2019) accomplished. Accordingly, a boronic acid COF was covalently linked to the outside of an amine-iron MOF (Figure 15). The original MOF is highly hydrophilic, but after covalently linking the COF to its outer shell, the nanocrystals become hydrophobic, as confirmed by water contact angle measurements. This new core-shell hybrid material can be used as a heterocatalyst that oxidizes styrene selectively and efficiently. Peng et al. (2018) have also adopted this version of “graft-to” using the MOF core and using interfacial polymerization create a COF shell through imine condensation reactions. This new MOF-COF shell hybrid shows a potential for visible-light-driven photocatalyst for the degradation of organic pollutants. Chen et al. (2020) synthesized another example of a MOF-COF core-shell. In this work a hydrazone-linked COF (TFPT–DETH) was synthesized *in situ* on the surface of octahedral NH_2_-UiO-66. This new hybrid showed excellent hydrogen evolution potential as a photocatalyst.

#### 2.2.3 Graft-with

“Graft-with” describes a simultaneous formation of a polymer and a POF, where they grow together in a “graft-with” manner. This approach could lead to a tightly integrated composite material with unique properties derived from both components. In this process, the monomers of the polymer, the metal ions or clusters, and the ligands on the metal species need to be introduced either simultaneously or sequentially, allowing them to react and form bonds concurrently. This can be achieved by carefully selecting reaction conditions—such as temperature, solvent, and catalysts—and by precisely choosing the reacting materials, including metals, monomers, and ligands. The resulting material would have characteristics of both the polymer and the POF, potentially offering synergistic properties. For example, the polymer component could provide flexibility and mechanical strength, while the MOF component could contribute to high surface area and porosity. Applications for such composite materials could range from gas storage and separation to catalysis and sensing, depending on the specific properties of the resulting material. The key to success would be to optimize the reaction conditions to control the growth and integration of both components and understanding how their properties interact on a molecular level.

**FIGURE 16:**
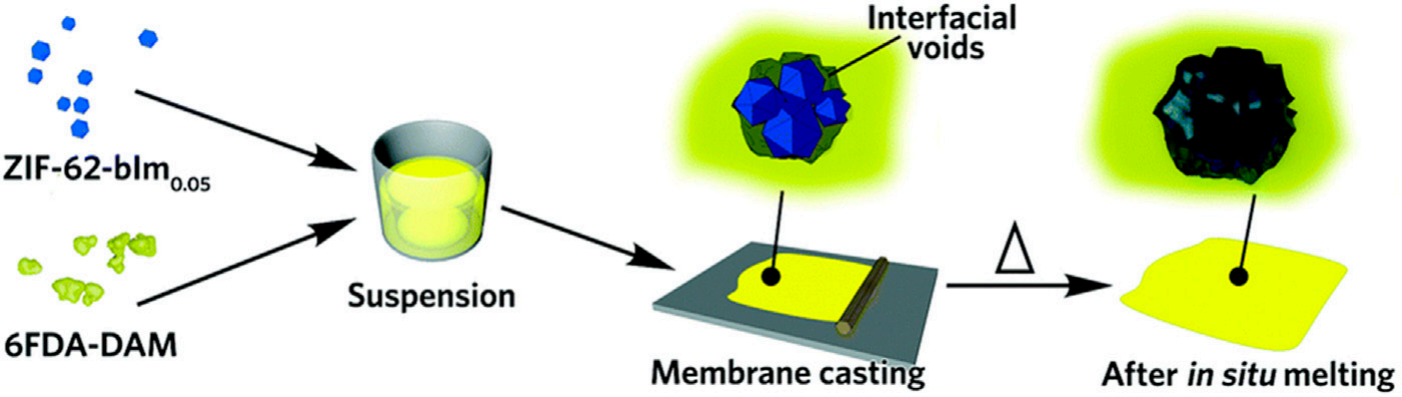
“Scheme of the preparation of glass ZIF-based mixed-matrix membranes.” Published with permission (Lin et al., 2020).

Although this polymerization category, “graft-with,” has not yet been accomplished there are similarities to current work which mostly include incorporating a POF into a polymer membrane. For example, Lin et al. used a zeolitic imidazolate framework (ZIF) as the filler for a 6FDA-DAM polyimide-based composite membrane which resulted in an impure membrane with interfacial voids (Figure 16). After *in situ* melting, the membrane was annealed, transforming it into a glass plate and effectively filling the voids to enhance its structural integrity and performance as a membrane as shown by TGA and XRD (Lin et al., 2020). Similarly, Marti et al. (2018) *in situ* synthesized a zirconium-based MOF while simultaneously curing the Matrimid polymer matrix. By changing the MOF loading percentages within the mixed membrane the gas separations of N_2_/CO_2_ were enhanced compared to the MOF alone.

The *in situ* formation of a COF in the presences of a polymer membrane through fixed interfacial polymerization is also an emerging field. Zhao et al. (2022) have integrated a COF and a POP (Porous Organic Polymer) into a polypropylene separator. This separator acts as a semi-permeable membrane, effectively dividing the organic and aqueous phases and creating an interfacial junction. With this new COF-Separator membrane battery performance testing was done by manufacturing a Li-S coin cell. Cyclic voltammetry was employed as the primary analytical technique to assess the battery’s potential and evaluate the durability of the material over multiple cycles.

##### 2.2.3.1 Summary

**FIGURE 17:**
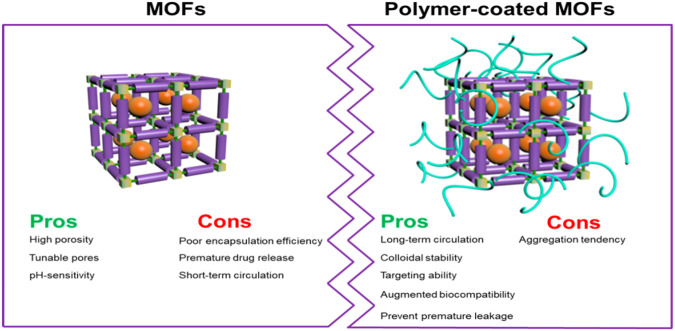
“Schematic illustration the combination of polymer and MOF and their pros and cons.” Published with permission (Zhong et al., 2019).

In summary, there are many advantages of adding a polymer to the surface of a POF as shown in (Figure 17). When incorporating polymers on the surface of a POF, there are three primary categories of polymerization, graft-from, graft-to, and graft-with. “Graft-from” describes a POF acting as an initiator to start the polymerization process of a monomer, which ultimately creates a homopolymer on the surface of a POF. This form of polymerization normally requires intermolecular interactions such as hydrogen bonding, electrostatic properties and p-p stacking between the polymer and the POF surface. “Graft-to” involves attaching pre-formed polymer chains to the surface of the POF through covalent bonds. Pre-formed polymer chains covalently bond onto the surface by reacting the polymer end-groups with complementary functional groups present on the surface. It offers precise control over the placement of polymers but can be limited by steric hindrance as the surface becomes crowded. “Graft-with” is a less explored category, which involves the simultaneous formation of the polymer and the POF, leading to a more integrated and potentially synergistic composite material. The polymer and POF grow together, creating a highly interwoven structure.

### 2.3 Polymers among POFs

POFs and polymers are both supramolecular structures of repeating carbon-based units but possess very different mechanical properties. Polymers are typically soft and processable, POFs are generally hard, brittle, granular materials that are difficult to process. The incorporation of POFs with polymers allows for the utilization of unique and differing properties of each material. The chemical linking of POF to polymer is a powerful technique to incorporate these two materials. To achieve this route, polymer and POF need available functional groups compatible with one another for covalent or coordination connections. The functionalization of POFs or polymers can occur pre or post polymerization allowing for a variety of synthetic pathways. POFs can be fabricated directly to the backbone of polymer chains, using them as a template and scaffolding to create hybrid materials. Through ligands, POFs can be integrated along the backbone of polymers to create composite materials with capabilities of changing the polymer’s original morphology. The linking of materials is not, however, solely limited to chemical connections.

#### 2.3.1 Polymerization among POF particles

POF nanoparticles with polymerizable functional groups offer the advantage of integrating them into polymers. This integration allows for the creation of hybrid materials, exploiting the properties of each material and allows for advantageous properties of both polymers for materials with enhanced functionality and rigidity. Polymerization takes place between functional groups of the POF particles and polymers. For this to happen, the POF particles need to have polymerizable groups. The interactions that can be exploited between monomers and functional groups vary from strong interactions such as covalent bonds and dynamic coordination complexation to weak interactions such as hydrogen bonds, π-π interactions and halogen bonding (Beuerle and Gole, 2018). These chemical interactions bring together small and rigid subcomponents to form dynamic supramolecular structures with distinct chemical and physical properties for next-generation materials. The number and spatial arrangement of functional groups and the molecular building units can form connected cage networks or infinite porous frameworks both in the 2-dimensional (2D) and 3-dimensional (3D) regime (Beuerle and Gole, 2018).

The subsequent supramolecular structures can be examined by three hierarchy levels. The molecular level, which contains the functionality and code for the desired framework geometry and topography. The assembly level, which involves the construction of the large molecular structure, how uniform and repeating the structure is, the dimensions of the structure, and how the structure interacts and orientates itself. The final level of these materials is processing for applications and utilizing their functional properties (Beuerle and Gole, 2018). The molecular level is the foundation when designing and preparing polymer-POF hybrid materials. The use of ligands that contain polymerizable groups to form POF materials is an extremely powerful technique that allows for increased processability, which is typically a huge limitation of these materials.

**FIGURE 18:**
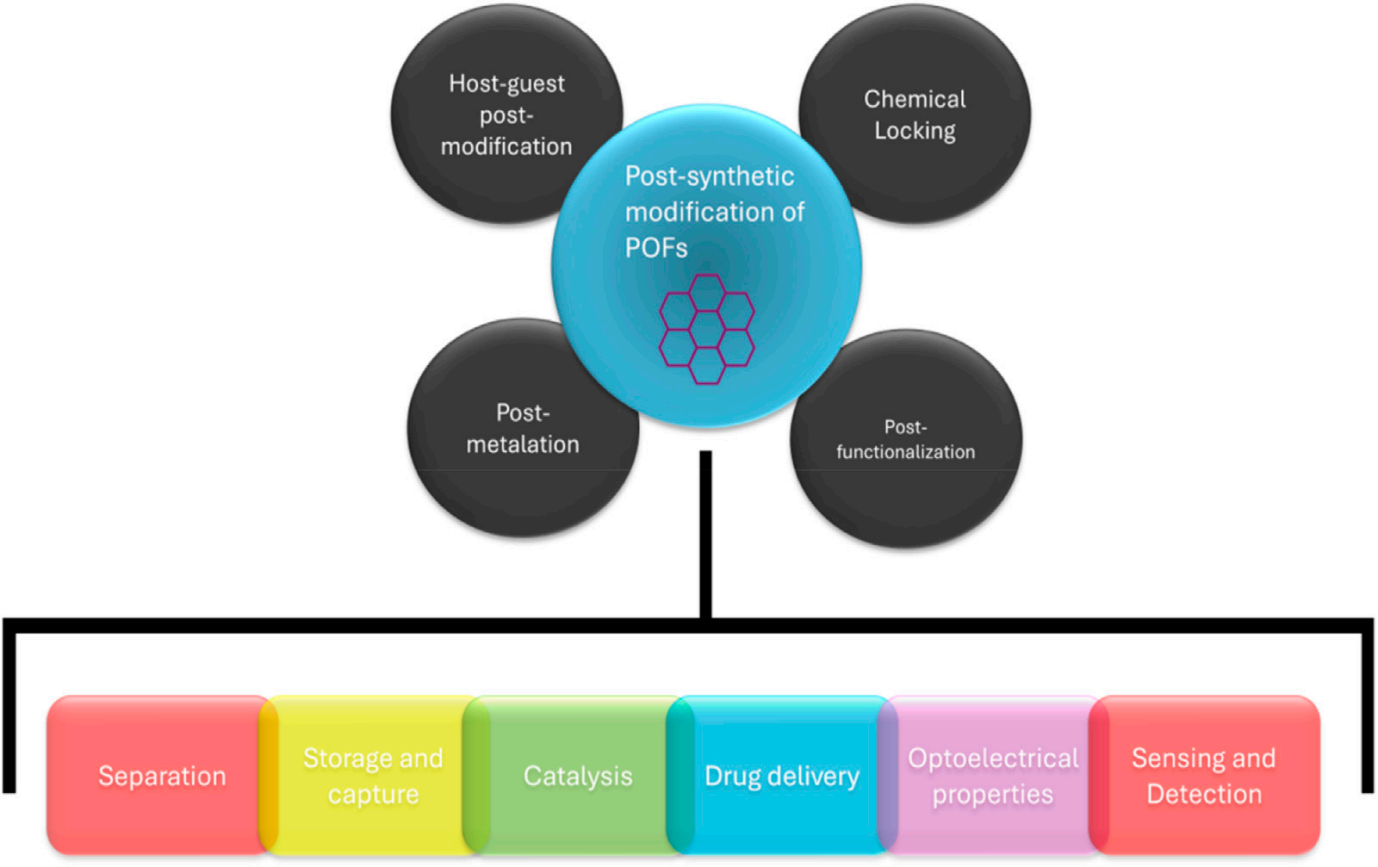
Various post synthetic modification strategies and with their potential application (Rejali et al., 2023).

There are two different methods for the formation of Polymer-POF hybrid materials where polymer is among POF particles (Figure 18). Ligands for POFs can be synthetically modified with functional groups before polymerization; or a post-synthetic modification (PSM) can be done, where the POF material is modified with functional groups post polymerization (Lyle et al., 2019). This modification allows for the functionalization of the generated material, as well as a linkage site for polymer-POF hybrid generation. Functionalization of monomers before polymerization is a good technique when desiring homogenous functionality. Small molecules like ligands are much easier to characterize than large molecular structures, and the degree of modification can be precisely determined before polymerization takes place. PSM is a useful technique when the addition of functional groups requires harsh synthetic environments where the ligand is not stable, but the subsequent robust framework is. PSM can be achieved through covalent and coordination chemistry, allowing for the incorporation of POFs into polymers for robust hybrid materials (Rejali et al., 2023).

Cohen *et al* synthesized a nylon-MOF composite using a post-functionalization strategy by reacting adipoyl chloride with a NH_2_ functionalized MOF producing acid chloride end groups. The modified acid chloride MOF was then polymerized with hexamethyleneidamine (HMDA) *via* a interfacial polymerization to form a nylon composite (Kalaj et al., 2019). Cook and Rzayev *et al* synthesized a polymer-MOF hybrid by covalently integrating poly (amic acid) and MOF-5 structure using ligand exchange between pure MOF crystals and ligand moieties incorporated into the backbone (Pastore et al., 2018). The result of the integration was a cross-linked composite that maintained the functional properties of MOF-5 but with the increased strength, flexibility, and stability of a traditional linear polymer.

Wang and co-workers created a composite membrane for dye rejection using an interfacial polymerization technique where COF nanosheets are deposited on a microfiltration membrane support. A polyimide COF thin film is created on top of the support. The integration of the porous material with the polysulfone support resulted in enhanced water permeability with high dye rejection capabilities. The researchers found that adjusting the COF loading and monomer concentration has huge implications on the thickness of the thin film, which is a crucial factor in performance (Wang et al., 2020).

Including polymerizable groups into POFs provides many opportunities for creating advanced hybrid materials. Beyond creating hybrid materials, the addition of functional groups allows for better dispersion and more uniform composites, leading to increased homogeneous distribution which helps maintain integrity and performance of the composite material all while minimizing agglomeration of nanoparticles, which can often cause weak points in the system and hinder effectiveness.

#### 2.3.2 Functionalization of POFs with polymers

POFs are generally synthesized as crystalline, polycrystalline, or semi-crystalline powders. To utilize the many potential functionalities of these materials, they largely need to be processed to make them viable for application purposes. Incorporation of the POF material into a new system generally makes the handling and implementation considerably easier. The task of integrating POFs into systems or devices is not trivial and is a complex problem that has led to much interest in developing technologies with integrated POF materials. The porous materials are tailored with discrete pore sizes and chemical functionality; it’s important to recognize that the processing of these systems also has a high degree of tailorability, causing variations based upon the desired application, the method used for processing, and the chemical dimensions and makeup of the material.

One method of processing involves chemically linking with polymers that are easy to process. This is usually done as a postsynthetic polymerization (PSP), utilizing polymerizable functional groups on the POF material, and reacting to it, a linear and amorphous polymer. This copolymerizing helps with elasticity and processability of the material (Zhang et al., 2015a). There are several pathways for copolymerizing to occur, covalent bonding, which utilizes strong covalent bonds between reactive sights on the material and the polymer, such as (1) coordination bonding, where either the POF, polymer, or added ligand has a coordination between the polymer and POF; and (2) hydrogen bonding and Van der Waals interactions, these are non-covalent interactions where bonds are formed through the attraction between hydrogen atoms, and the lone pairs of electronegative atoms. These bonds are less robust than covalent interactions but allow for reversibility and more uniform networks. Van der Waals interactions are weaker than hydrogen bonding yet play a pivotal role in dispersion and interaction between polymer and POF. The weaker forces between POF and polymer are particularly advantageous in applications where reversibility and self-healing are desired.

Zhang et al. (2015a) synthesized a self-standing membrane via PSM by functionalizing a MOF (UiO-66-NH) with methacrylamide groups, mixing it with butyl methacrylate (BMA) and a photoinitiator in suspension. After irradiating with UV light for several minutes, they synthesized an elastic and stand-alone membrane taking on the shape of the mold used. Kalaj and Cohen (2020) reported a similar technology in which they made a catalytically active material by spray-coating UiO-66-NCS tethered to an amine terminated polypropylene polymer onto nylon fibers. The MOFs were crosslinked into a MOF-polythiourea (MOF-PTU) composite material that retained the catalytic properties of the MOF and the flexibility of the polymer.

Another very closely related PSM to covalent linking, is coordinative linking. Where a polymer is coordinated between and links MOF particles (Kalaj and Cohen, 2020). Shimizu and co-workers, prepared a flexible MOF composite by covalent cross-linking with a metal-organic polyhedra. They confirmed MOF structural integrity through x-ray diffraction (XRD) and electron microscopy. The generated hybrid material showed an increase in the reduced elastic modulus and hardness as the amount of cross-linking was increased. This allowed them another degree of tailorability by varying the hardness and still maintaining original MOF’s properties (Lal et al., 2019).

Not only does processing of the POF material allow it to be incorporated into a system, but when incorporated, most of the limitations of these powdered materials are largely reduced (Lal et al., 2019). The benefits of copolymerizing and the formation of hybrid materials include enhanced chemical and structural rigidity, self-standing materials, and increased tunability of materials for a given application.

#### 2.3.3 POF formation along polymer chains

Formation of POFs using polymer chains with ligand end groups involves a sophisticated interplay of chemistry and material science generating materials with unique and tailorable structures and properties. This process utilizes the fundamentals of coordination chemistry and self-assembly molecules to generate hybrid framework materials arising from the merging of polymer chemistry and coordination chemistry. Metals on the ends of the polymer chains coordinate with ligands and metals of other polymer chains interlinking and forming long range structures. These subsequent connections form an extended network of polymers and metals. An example of this is polymer metal-organic cages (polyMOCs).

PolyMOCs are generated by the polymerization of terminal metal ions and organic ligands into discrete, 3-dimensional, cage-like structures connected by polymer chains. The metal ion is the linker, connecting cages together and creating a network of MOCs. Polymer chains with ligands on the ends come together and the ligands form a porous framework. As polymerization occurs, the polymer chains become entangled with one another, resulting in a stable, hybrid material and combines the properties of the polymer chain and metal-organic cages; in the case for polyMOC materials. As with all composite materials, the choice of starting materials and copolymer dictate the properties and performance of a generated material.

**FIGURE 19:**
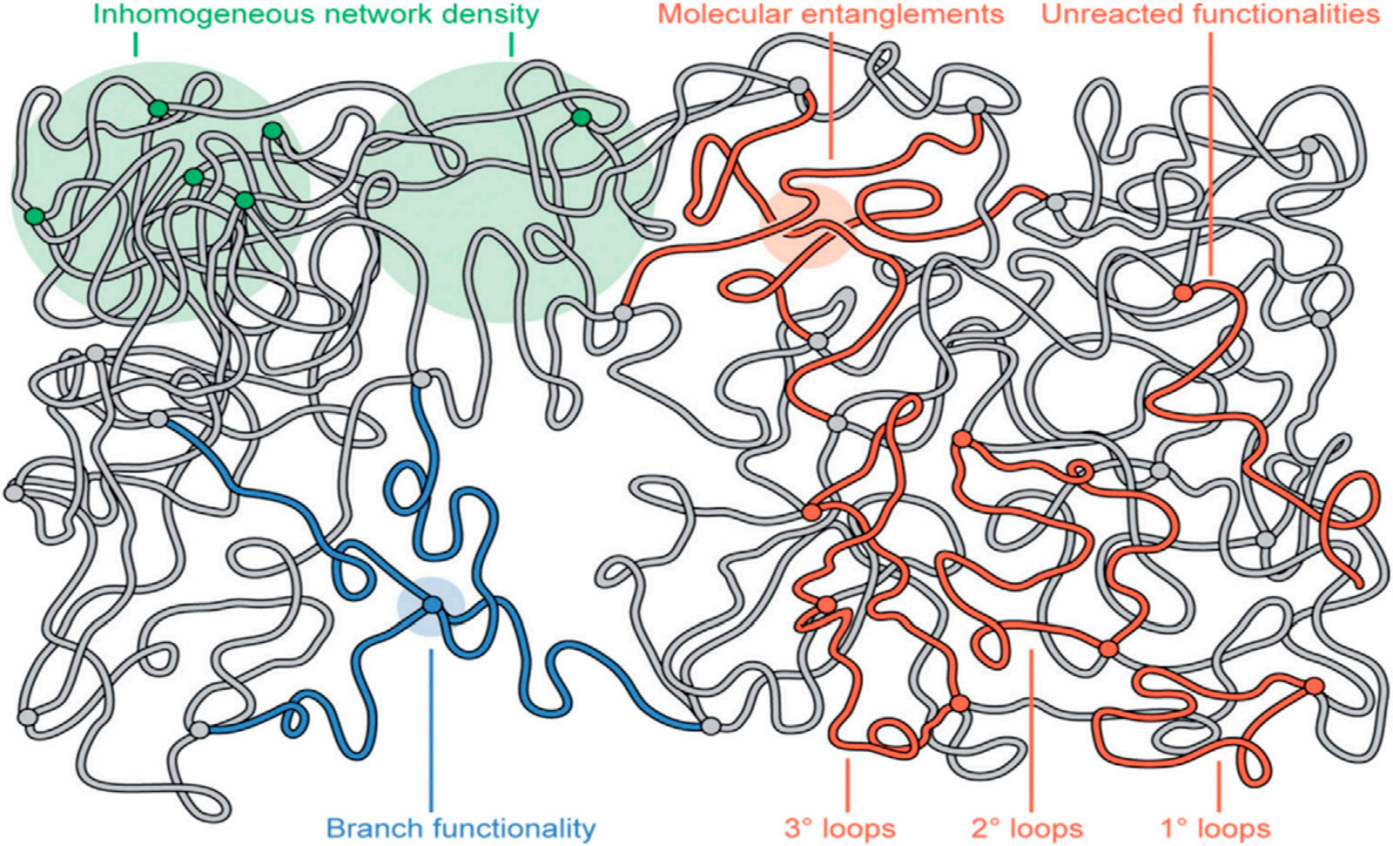
The complex interactions that take place within linear amorphous polymers. Interactions can be categorized based upon length scales of 10–100 nm (green), 1–10 nm (red), and <1 nm (blue) (Gu et al., 2020).

Gu et al. (2020) were able create an array of polyMOCs using the same components but varying the concentration of each component. The three-component system included a polymer terminated with ligand ends, a small ligand molecule, and a palladium ion. They reported effects such as different network formation, mechanical properties, dynamics, and functionality when varying the concentration of the palladium ion and small ligand molecule; the polymer concentration remained constant throughout the studies (Figure 19). The main finding of the article was the control over tunability of one system and being able to precisely control the selected properties based upon starting reagent concentrations. Johnson and different co-workers were able to do further vary structural design by using a Cu_24_L_24_ polyMOC and performing solution-gel transition by irradiating with UV light (Wang Y. et al., 2017). The Cu oxidation state was varied under UV irradiation and with the help of a photosensitizer; the reversibility of the Cu was able to switch between Cu^2+^, Cu^+^, and Cu^0^. Cu+ and Cu^0^ states lead to the disassembly of the hybrid network and form a solution. Once oxidized to Cu^2+^ the system complexes and forms supramolecular gelation (Wang Y. et al., 2017).

The ability to tailor different phases and structures in one system lends a high level of processing and tuneability. Nitshcke and co-workers report a polyMOC hydrogel having two internal phases that release small molecules at different rates (Oldenhuis et al., 2020). Rapid self-assembly allows the formation of hydrogel microparticles, which have a dynamic response to stimuli and a controlled release (Oldenhuis et al., 2020). The varying internal phases lead to different release rates based on their encapsulation within the cage’s internal phase. This method enhances host-guest chemistry for applications including drug delivery, catalysis, and sensing, offering a versatile platform for multifunctional materials development.

The ability to introduce stimuli-responsive behaviors and enabling polyMOC transitions between differing physical states, sol-gel transitions as an example, or alter the network configuration from an environmental change is a small window into the potential these materials have in any given application. The increase in potential applications is largely spearheaded by the enhanced processability that comes with such properties. Being able to control phase helps when processing a material with poor dispersibility and conglomerates formation in a polymer solution. Consequently, the combined ability to functionalize the material and the fabrication process in a highly controlled manner is less like a doorway and more of a gateway into the vast expanse of possibilities.

#### 2.3.4 Polymer chain with ligands

Polymers and coordination molecules can not only form linked cage-like materials, but they can also be utilized to connect polymers and POFs. MOFs can be hybridized using traditional amorphous or semicrystalline linear polymers by utilizing the transformation of polymer ligands directly into crystalline frameworks through the incorporation of metal ions (Bentz et al., 2020a).

It is important to note the effect of the complex interactions that take place between polymer chains themselves which can change the properties of the composite material. These interactions dictate physical, chemical, and topological properties. Physical interactions encompass non-covalent interactions such as van der Waals forces, hydrogen bonding, and electrostatic interactions.

Chemical interactions involve covalent interactions and bonding between functional groups. These interactions can be used for grafting or cross-linking. By specific control of conditions, the degree to which functional groups are utilized can vary. This will change the mechanical strength of the polymer as well as its functionality, impacted by the amount and nature of the unreacted functional group.

There are also topological interactions, which are the spatial arrangement and entanglement of polymer chains within the hybrid system. Interactions between polymer chains can lead to inhomogeneous polymer density within the system, generating areas of high and low polymer density, changing the strength and porosity of a material. Entanglement of polymer chains and the formation of coordination bonds with metal ions forms a robust and interconnected system. Entanglement enhances the mechanical and strength of these polymerized materials, including tensile strength, flexibility, and durability.

There are many linear, amorphous, non-porous polymers that can serve as ligands for MOF synthesis to form polymer-MOF hybrids. These hybrid materials exhibit the properties of MOFs and the polymer utilized for framework interlocking (Ayala et al., 2019; Lal et al., 2019). Matrix-mixed membranes are an example of connecting POF materials into a solid, flexible polymeric material by incorporating rigid materials into flexible polymers (Kalaj et al., 2020). Mechanical interlocking by polymer chain entanglement is a topological aspect of complex composite systems. There is evidence that copolymers can have a larger role than simply allowing for the processability of these composites. Cohen and co-workers synthesized a block co-polyMOF (BCPMOF) with controlled morphologies. BCPMOFs containing poly (1,4-benzenedicarboxylic acid) and morphology direction poly (ethylene glycol) (PEG) or poly (cyclooctadiene) blocks were used for the preparation of BCPMOFs. These copolymers were used to create hybrid materials with MOFs. They report that the architectures and weight fractions of the block copolymers significantly impacted the morphology of the resulting hybrid materials, allowing for control over particle size and shape (Ayala et al., 2019).

It is important to note that the blending of polymers to form these hybrid systems brings a variety of its own challenges, such as integration and integrity of the POF in the composite, which can hamper properties of the original materials (Lal et al., 2019). For instance, it is desirable to have a composite material with uniformly dispersed POF material. If there is lower interaction between the POF and polymer, aggregates are likely to form, causing areas with high and low POF and polymer density. Another issue caused by an un-uniformly dispersed load phase is comptonization of the POF functionality (Lal et al., 2019). Compression caused by the polymer onto the POF can alter the pore size and shape, changing the original properties. Linear polymers are typically employed for these composite systems and can fit and integrate into the polymer pores, altering the effective pore size of the POF. In membrane separation, a support is usually introduced to increase the strength of the composite membrane. If the load phase does not adequately adhere to the support, and exfoliates off, the lifespan of the composite is reduced, and the original abilities could be lost entirely (Lal et al., 2019).

Uemura and co-workers altered the configuration of flexible polymeric MOFs by the insertion of polymeric guest molecules. The incorporated guest polymers prevent the closing of the host framework and create a stable open form. Polymer chains cause an opening of occupied nanochannels, propagating to neighboring nanochannels and becoming accessible for adsorption. The hybrid materials were formed through *in situ* polymerization and resulted in homogenous loading of material, which greatly increased the MOF’s porosity. By limiting the possible structural configurations of the framework, the possibility for reactive intermediates was reduced as evidenced by the 200°C increase in thermal degradation temperature (Le Ouay et al., 2017). Zhang et al. (2015a) used various linear polymers functionalized with carboxylic acid groups for Zn^2+^ coordination. The amorphous linear polymers functioned as a polymer ligand upon annealing with Zn^2+^ to produce crystalline polymer-metal-organic frameworks (polyMOFs).

Polymer chains terminated with ligands are a powerful tool in the development of hybrid materials. Their ability to form complex structures through coordination with metal ions with enhanced mechanical properties that can be tailored and tuned for a specific application have seemingly limitless advantages over traditional single polymer systems.

In summary, the integration of POFs with polymers creates advanced hybrid materials that combine the unique properties of both components. The interplay between POF and polymer has many avenues through which composite materials can be generated, each with their own distinct advantages and disadvantages and changes to the material’s performance.

## 3 Characterization and properties of polymer-POFs

Polymer-POFs hybrid materials exhibit complex structures with diverse chemical compositions and porosities. Characterization techniques play a crucial role in understanding their chemical properties, morphology, and performance in various applications (Cote et al., 2005). Here we briefly provide an overview of the key characterization techniques employed for polymer-POFs in which typically three main types are used: radiation absorption using NMR and IR; microscopy techniques such as TEM and XRD and physical phenomena using TGA and BET.

### 3.1 Radiation absorption techniques

#### 3.1.1 Nuclear magnetic resonance (NMR) spectroscopy

**FIGURE 20:**
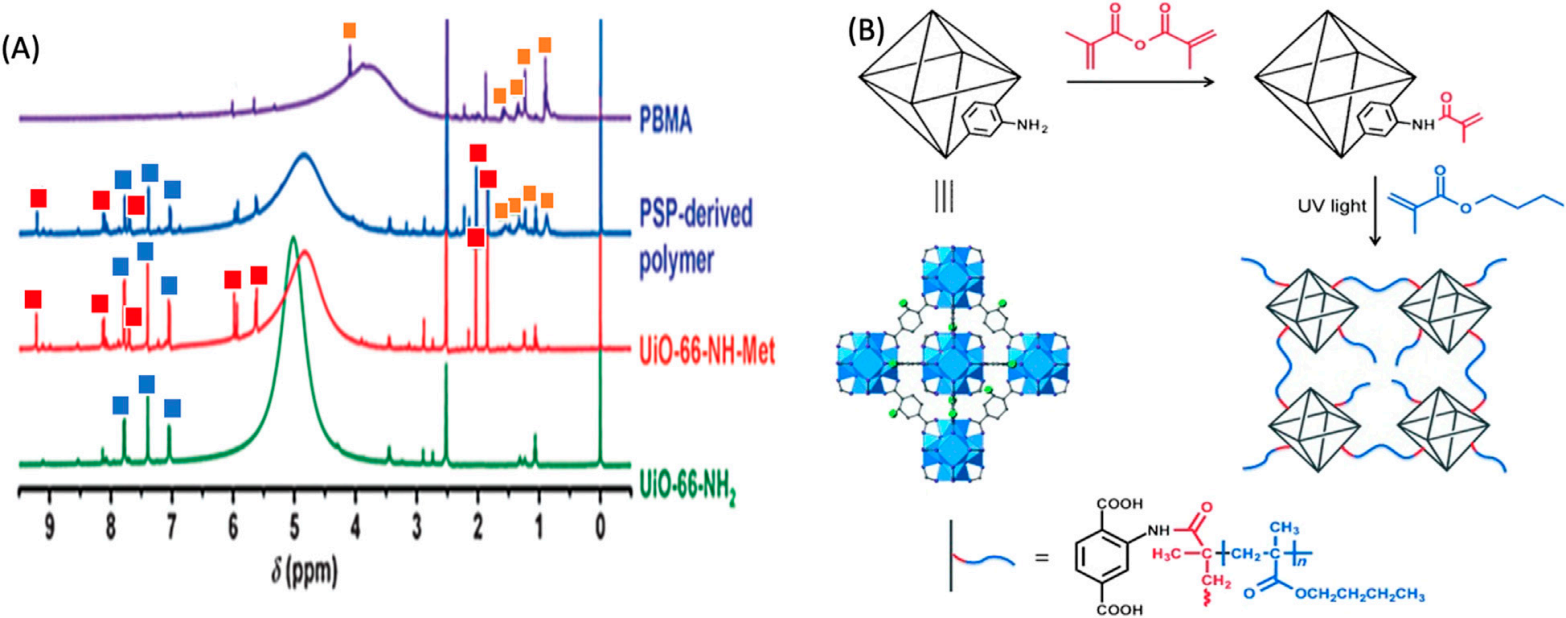
**(A)** 1H NMR spectra of degraded UiO-66-NH2, UiO-66-NH-Met, PSP-derived polymer, and PBMA. **(B)** Post synthetic modification of UiO-66-NH2 with methacrylic anhydride and subsequent polymerization with butyl methacrylate (BM A) by irradiation with UV reprinted with the permission (Zhang et al., 2015a).

NMR spectroscopy is utilized to investigate the molecular structure, dynamics, and chemical environment of polymer-POFs. By subjecting the sample to a strong magnetic field and analyzing the nuclear spin interactions, NMR provides information about molecular connectivity, conformation, and mobility, aiding in elucidating polymer chain architecture and cross-linking (Beuerle and Gole, 2018). In the modification of UiO-66-NH_2_, ^1^H NMR spectroscopy was used to confirm the structure (Figure 20A). Modified samples were degraded in a solution of HF in DMSO and new resonances at 2.0, 5.6, 5.9, 7.7, 8.1, and 9.2 ppm, which are consistent with the anticipated modified molecular ligand counterparts (Figure 20B) as calculated from the spectrum was estimated to be 67% (Zhang et al., 2015a). Zhang et al. (2015a) concluded that postsynthetic polymerization (PSP)-derived membrane overcomes all drawbacks of the aforementioned membranes. First, the incorporation of MOFs into the polymer brings in porosity and channels with a polarized surface, while the flexibility and processability of the polymer are maintained. Second, nanosized MOF particles with polymerizable functional groups are well dispersed and covalently anchored in the polymer without particle aggregation, and the formation of nonselective voids within and/or between the aggregates is thus avoided. Third, the copolymerization of MOFs and monomers contributes to the close interaction between particles and polymers, thus achieving homogeneity at the molecular level.

**FIGURE 21:**
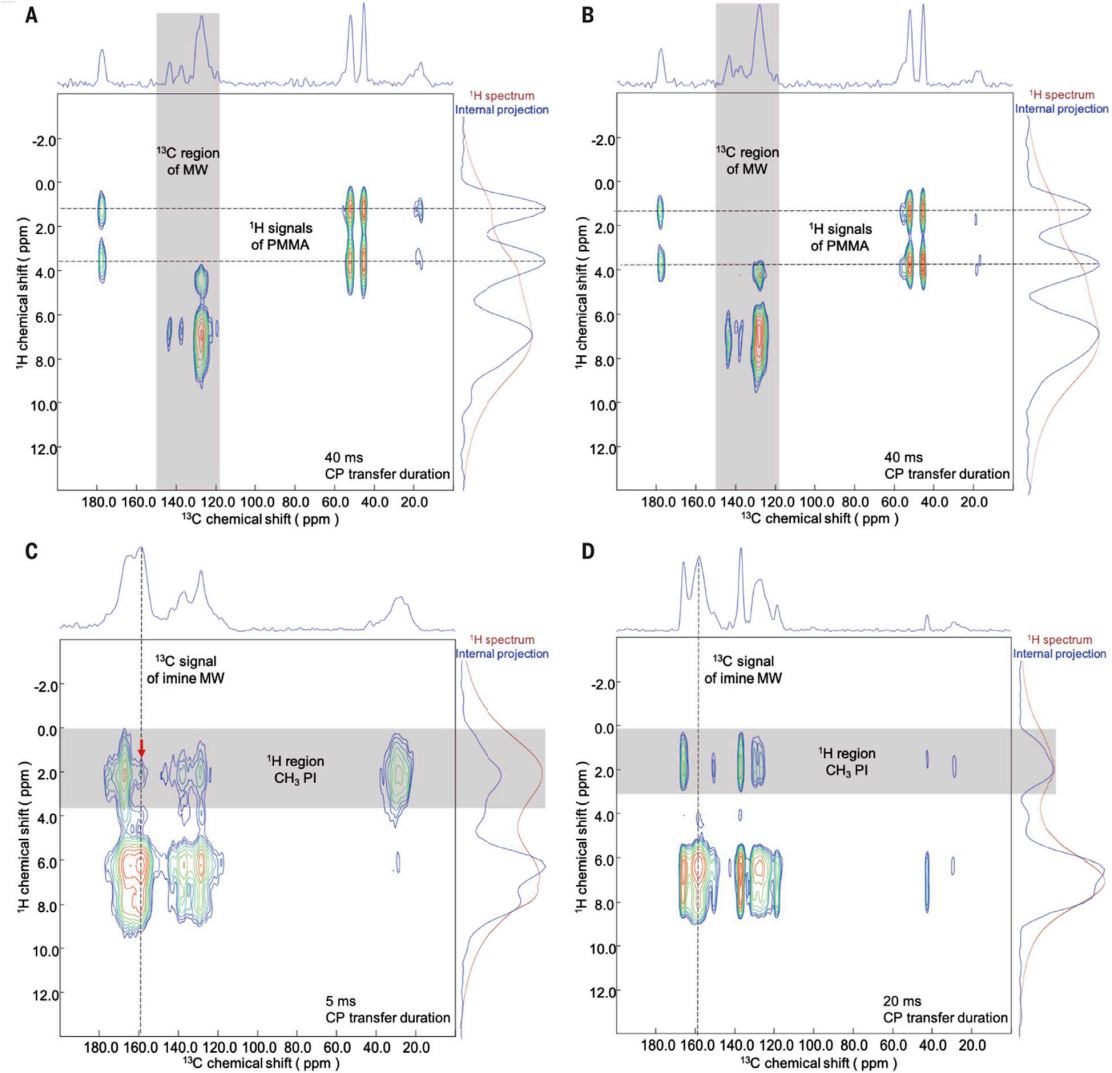
Investigation of polymer-COF interactions by CP-HETCOR solid-state NMR spectroscopy. **(A, B)** ^1^H-^13^C CP-HETCOR NMR spectra of a PMMA-MW (20:80 by weight) composite **(A)** and a PMMA-MW (20:80 by weight) physical mixture **(B)** showing no correlation spots between the polymer and the COF at a contact time of 40 ms. **(C)** ^1^H-^13^C CP-HETCOR NMR spectrum of a PI-MW (50:50 by weight) composite showing a clear correlation spot (red arrow) between the polymer and the COF at a contact time of 5 ms. **(D)** ^1^H-^13^C CP-HETCOR NMR spectrum of a PI-MW (50:50 by weight) physical mixture showing no correlation spot between the polymer and the COF at a contact time of 20 ms. permission reprinted (Neumann et al., 2024).

Yaghi and co-workers reported the nature of polymer-COF interactions using 2D solid-state NMR with cross polarization based heteronuclear correlation (CP-HETCOR) spectroscopy as depicted in Figure 21 (Neumann et al., 2024). This technique allowed them to probe the intermolecular proximities between the backbone of the COF and the polymer threads of PMMA and PI. Previous studies have demonstrated various correlations between surface interactions and polymer threading in polymer-MOF systems. In this study, they compared PMMA-MW and PI-MW samples with physical mixtures of the polymers and MW.

The CP-HETCOR technique requires sufficient resolution in both the ^1^H and ^13^C dimensions, as well as the presence of a unique correlation spot arising from a heteronuclear pair between the polymer and the COF. For PMMA-COF composites, the proton and carbon signals of the methyl and methoxy groups in PMMA were easily distinguishable from the predominantly aromatic backbone of the COF. CP-HETCOR experiments on PMMA-MW composites and physical mixtures, with long CP-transfer durations (contact times) of 40 ms, showed no correlation between the carbon and proton signals of the COF and PMMA. This indicated that the polymer was not in close proximity to the woven nanocrystals. These results provide evidence for a polymer-COF system dominated by surface interactions, where most of the polymer strands did not interact substantially with the pores of the COF.

#### 3.1.2 Fourier transform infrared spectroscopy (FTIR)

**FIGURE 22:**
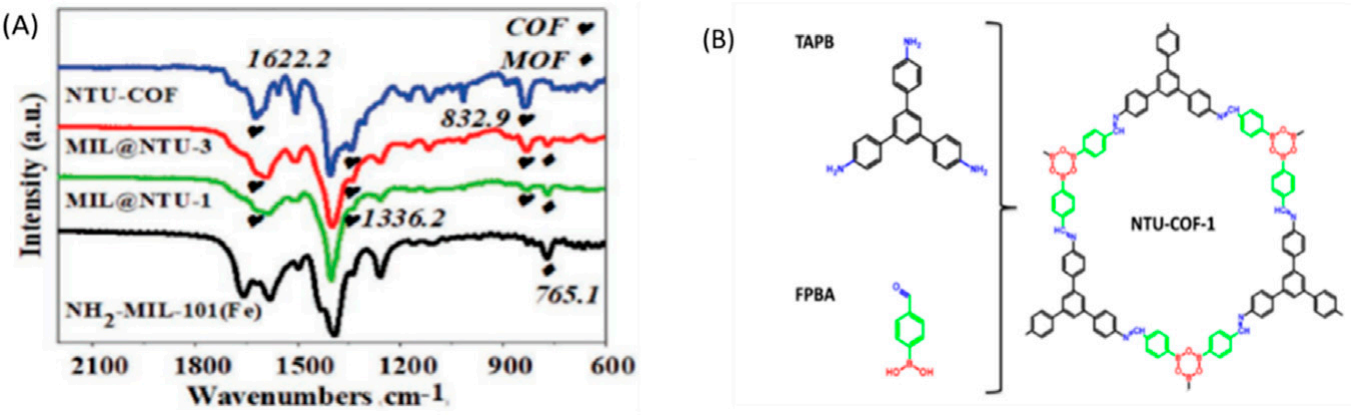
**(A)** FT-IR spectra of NTU-COF, MIL@NTU-1, MIL@NTU-3, and NH2-MIL-101(Fe), **(B)** The Structure of NTU-COF. Reprinted permission (Neumann et al., 2024).

FTIR is employed to analyze the chemical composition, and functional groups present in polymer-POFs. By measuring the absorption or emission of infrared light by the sample, FTIR produces spectra that reveal molecular vibrations characteristic of specific chemical bonds, aiding in identifying polymer chains and incorporated functional groups (Côté et al., 2005). Conformation of both NH_2_-MIL-101(Fe) and NTU-COF (Kandambeth et al., 2019) were achieved using FT-IR. The FT-IR spectrum of the core–shell structure matched well with the proposed structure (Figure 22). New stretches at 832.9, 1,336.2 (B-O), and 1,622.2 cm^−1^ (C=N) confirmed the formation of NTU-COF (Cai et al., 2019; Li et al., 2022).

### 3.2 Microscopy techniques

#### 3.2.1 X-ray diffraction (XRD)

**FIGURE 23:**
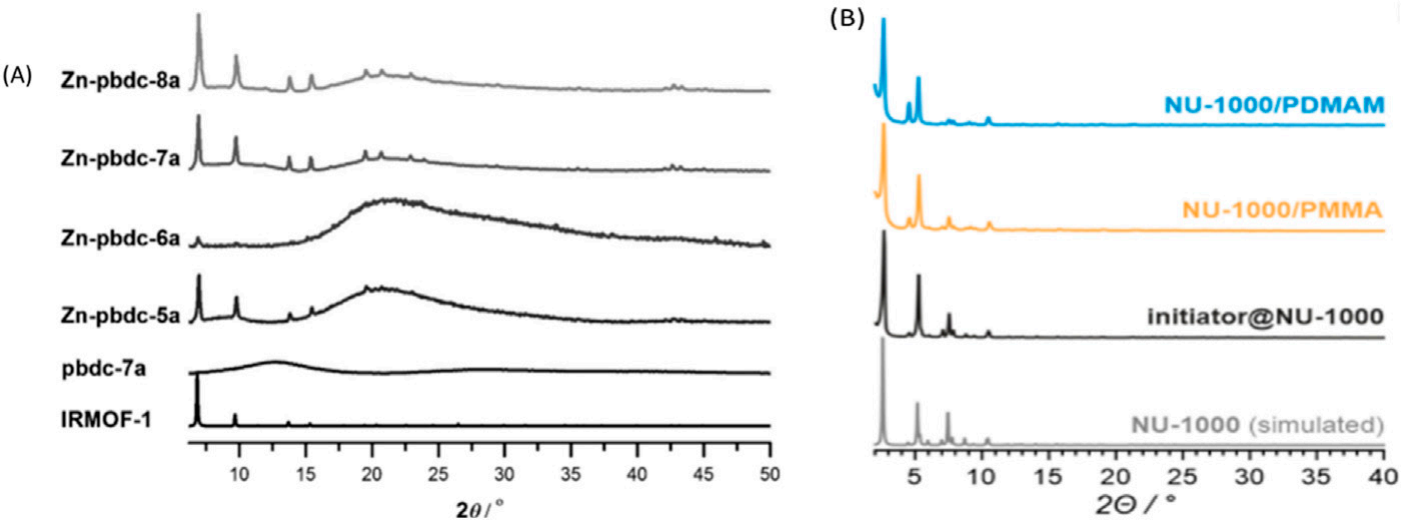
**(A)** PXRD patterns for pbdc-7a polymer ligand, Zn-pbdc-xa poly-MOFs (synthesized at 100°C), and calculated IRMOF-1. Reprinted permission (Zhang et al., 2015b). **(B)** Displays the PXRD of a polymer incorporated into Nu-MOF with sharp angles of the initiator@NU-1000, NU-1000/polymer and pristine NU-1000 materials. Reprinted permission (Pander et al., 2023).

XRD is employed to determine bulk crystallinity and phase composition of polymer-POFs. By analyzing the scattering pattern of X-rays interacting with the material’s atomic structure, XRD provides information about crystallographic orientation, lattice parameters, and presence of amorphous regions (Côté et al., 2005; Furukawa et al., 2013). For example, as synthesizing Zn-pbdc MOF (Figure 23A), its PXRD patterns of all of the products exhibited reflections that indicated the formation of an IRMOF-like network. All of the PXRD patterns also showed a broad peak centered at *2θ ≈ 22* ^*0*^, also indicating the existence of an amorphous phase (Zhang et al., 2015a).

In comparison, Figure 23B displays the PXRD of a polymer incorporated into Nu-MOF with sharp angles.

#### 3.2.2 Scanning electron microscopy (SEM)

**FIGURE 24:**
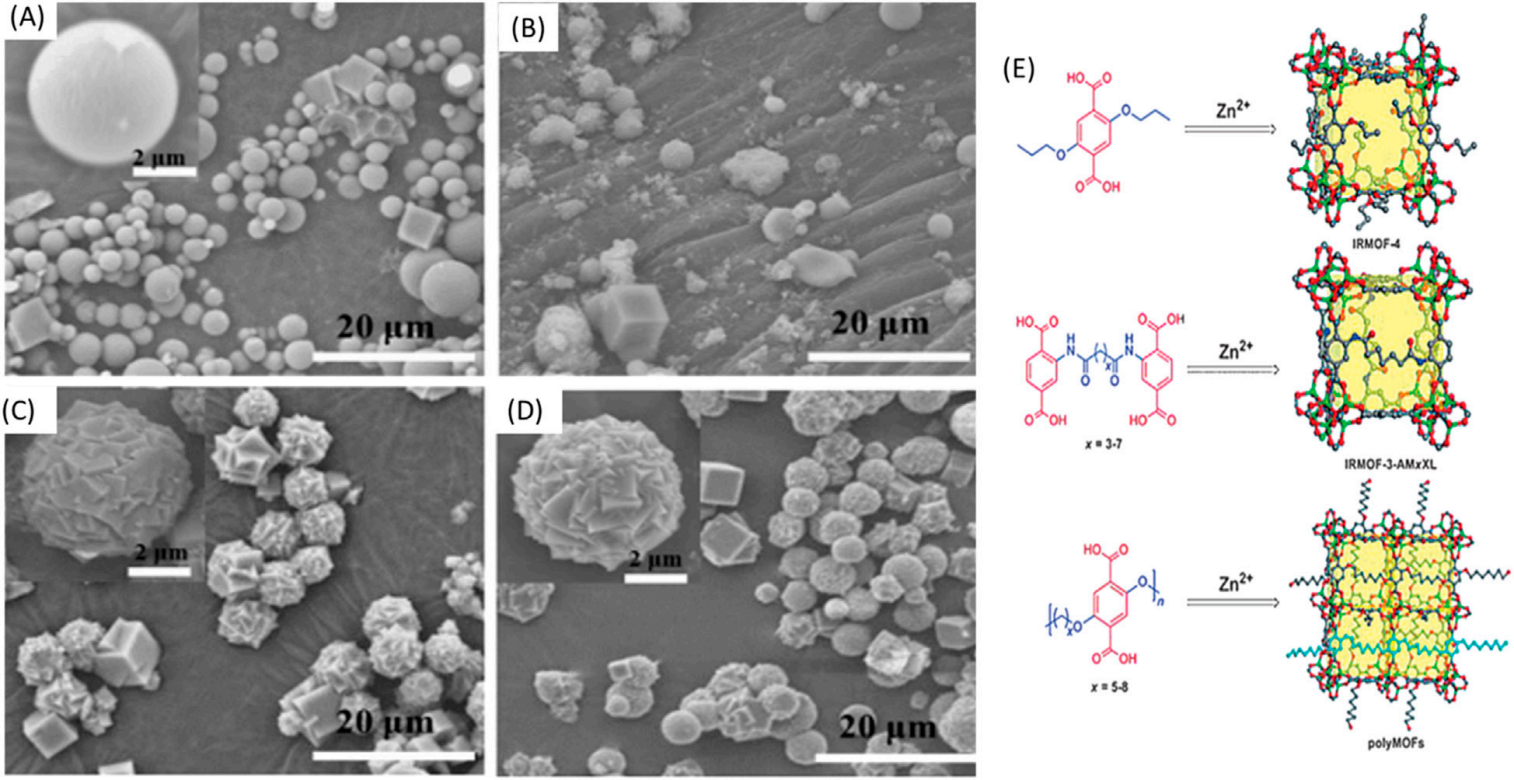
**(A–D)** SEM images of Zn-pbdc-xa MOF: **(A)** Zn-pbdc-5a; **(B)** Zn-pbdc-6a; **(C)** Zn-pbdc-7a; and **(D)** Zn-pbdc-8a. **(E)** The evolution of IRMOF derivatives constructed from (top to bottom): an H2bdc ligand derivative, a cross-linked H2bdc ligand, and a polymeric H2bdc polymer ligand. One polymer chain segment in the bottom image is highlighted in cyan for clarity. C gray; O red, Zn green. Reprinted permission (Zhang et al., 2015a).

SEM is an often-utilized tool to investigate the surface morphology and pore structure of polymer-POFs at micro-to nano-scale resolution. By bombarding the sample with electrons, SEM generates high-resolution images, enabling researchers to visualize pore size, distribution, and overall morphology (Hirschle et al., 2016). Zhang and co-workers proposed that unlike typical IRMOF materials (for example, IRMOF-1), which form large, macroscopic crystals, the particle size of the polyMOF samples formed at 100°C was on the order of 1–10 mm. As shown in Figure 24, the majority of Zn-pbdc-5a particles possess a spherical with regular facets, (Zhang et al., 2015a; Zhang et al., 2017) which was interpreted as the polycrystalline, spherical superstructures of Zn-pbdc-7a and Zn-pbdc-8a as the result of many individual crystals growing together and partially sharing polymer ligands.

**FIGURE 25:**
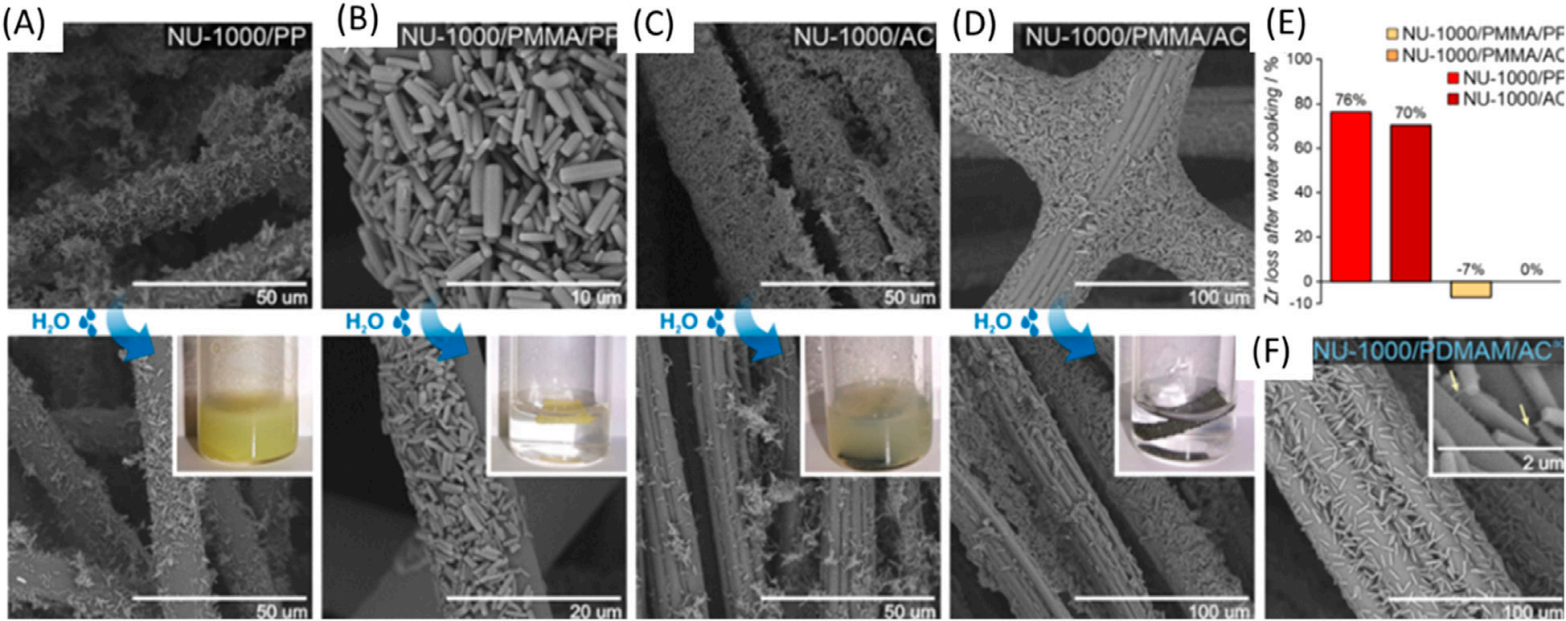
**(A)** NU-1000/PP, **(B)** NU-1000/PMMA/PP, **(C)** NU-1000/AC and **(D)** NU-1000/PDMAM/AC; **(E)** Zr loss (in %); **(F)** SEM image of the NU-1000/PDMAM/ACaq composite prepared using the aqueous dispersion of NU-1000/PDMAM. Reprinted Permission (Pander et al., 2023).

In studies on the impact of FRaP-in-MOF on MOF crystals attachment to textile fibers, SEM images of the MOF/fiber and MOF/polymer/fiber composites where observed before (Figure 25 top) and after (bottom) water treatment after soaking the composites in water. The arrows indicate the polymer phase involved in composite adhesion showing the process of reactions and structures of the composites (Pander et al., 2023).

#### 3.2.3 Transmission electron microscopy (TEM)

TEM is utilized to investigate the internal structure and morphology of polymer-POFs at nanometer scale. By transmitting electrons through thin sections of the sample, TEM produces high-resolution images, enabling visualization of internal pore structure, particle morphology, and interfaces, aiding in understanding material heterogeneity and nanoscale features.

Omar Yaghi and his co-workers demonstrated the dispersion of MW nanocrystals in a PMMA matrix through TEM imaging of PMMA-MW (3 wt%). The TEM images showed well-dispersed MW nanocrystals within the PMMA matrix. At this concentration, TEM analysis revealed that the interparticle distances in the PI-MW composite were approximately 1.7 (±1.2) mm (see Supplementary Figure S1). These observations collectively indicated that the effect of the polymer-COF junction extends beyond the typical range of filler-matrix interfaces, which is usually limited to tens of nanometers. This is in stark contrast to the catastrophic ruptures observed in pure PI, demonstrating the significant improvement in mechanical properties due to the incorporation of MW nanocrystals.

### 3.3 Physical phenomena

#### 3.3.1 Thermogravimetric analysis (TGA)

TGA is employed to study the thermal stability and decomposition behavior of polymer-POFs. By measuring the change in sample weight as a function of temperature under controlled atmospheres, TGA determines decomposition temperatures, thermal degradation kinetics, and residual mass, providing insights into material stability and degradation pathways (Xu et al., 2023; Sun et al., 2024). TGA profiles of NH_2_-MIL-101(Fe), MIL@NTU-1, MIL@NTU-2, MIL@NTU-3, MIL@NTU-4, MIL@NTU-5 and MIL@NTU (Cai et al., 2019) illustrate the thermal stability of the incorporated polymer MOF-COF matrix (Supplementary Figure S2).

#### 3.3.2 Gas adsorption analysis (BET)

BET analysis is employed to determine the surface area and pore size distribution of polymer-POFs. By measuring the adsorption of gas molecules onto the material’s surface at different pressures, BET analysis allows for quantification of specific surface area, pore volume, and pore size distribution, providing insights into porosity and accessibility of pores (Ding and Wang, 2013; Furukawa et al., 2013). Zn-pbdc-8a, Zn-pbdc-7a, IRMOF-L1, IRMOF-L2, and IRMOF-1 can uptake 41, 49, 30, 35, and 20 cm^−3^g^−1^ of CO_2_ at 1 atm and 298 K, respectively are illustrated in Supplementary Figure S3. The pore size distribution was calculated by density functional theory (DFT) from the N_2_ sorption isotherms. IRMOF-1 possesses the largest pore size (13Ǻ), IRMOF-L1 and IRMOF-L2 possess medium pore size (11Ǻ), and Zn-pbdc-7a and Znpbdc-8a exhibited the smallest pore sizes (7Ǻ and 9Ǻ). The reduction in pore size most certainly originates from the incorporation of polymer chains in the Zn-pbdc-xa frameworks, making the pore widths of the polyMOFs smaller than those of IRMOF-1, IRMOF-L1, and IRMOF-L2 (Zhang et al., 2015a; 2017).

Characterizing polymer-porous organic frameworks (P-POFs) requires a range of techniques to analyze their structure, porosity, thermal stability, and functional properties. Structural analysis often begins with X-ray diffraction (XRD) to determine if the material is crystalline or amorphous, providing insights into the crystal structure or the degree of disorder. Fourier-transform infrared spectroscopy (FTIR) is used to identify functional groups and confirm the framework’s formation by analyzing characteristic vibrational modes. Nuclear magnetic resonance (NMR) spectroscopy, both in solid and liquid states, offers detailed information on the local chemical environment, molecular structure, and dynamics. Porosity and surface area are assessed through gas adsorption techniques like BET and BJH, which measure surface area, pore size, and volume, while methods like small-angle X-ray scattering (SAXS) provide nanoscale pore information.

Thermal and mechanical stability is evaluated using thermogravimetric analysis (TGA), differential scanning calorimetry (DSC), and dynamic mechanical analysis (DMA), which provide data on decomposition temperatures, thermal transitions, and mechanical properties, respectively. The morphology of P-POFs is examined using scanning electron microscopy (SEM) and transmission electron microscopy (TEM) to assess surface texture and internal structure, while atomic force microscopy (AFM) evaluates surface properties. Chemical stability and functional characteristics are determined using X-ray photoelectron spectroscopy (XPS), elemental analysis, and inductively coupled plasma mass spectrometry (ICP-MS). Application-specific characterizations include studies on gas adsorption, catalytic activity, and sensing capabilities, tailored to evaluate the performance of P-POFs under specific conditions.

#### 3.3.3 Computational modeling and machine learning

The virtually limitless number of molecular combinations that can generate promising materials is one of the greatest boons to the fields of polymers and POFs, but also one its greatest challenges. This challenge is only amplified when the separate materials are combined. To explore this design space synthetically would be an insurmountable task. On the other hand, computational approaches are well suited to design hypothetical materials and screen for their compatibility, stability, and performance at a much faster rate than is experimentally possible. Between the significant advances in computational power and the advent of machine learning (ML), our understanding of fundamental molecular interactions and materials-by-design is rapidly expanding. The following section briefly summarizes the state of computational work in the individual fields of POFs and polymers—first focused on traditional atomistic modelling methods and then on ML—in order to contrast the challenges and current state of modelling polymer-POF hybrid materials, which is the primary focus of this review.

Quantum mechanical (QM), or *ab initio,* calculations rely on the Born-Oppenheimer approximation which allows the separation of the electron wavefunction from the nucleus wavefunction due to the substantial differences in mass, and therefore momentum, between the two particle types. Despite the approximations made by *ab initio* in order to solve the Schrödinger equation for the electron wavefunction, these calculations remain very computationally expensive and can be prohibitive depending on system size. Although computationally expensive for large unit cells, due to the periodic crystalline structure of POFs, *ab initio* methods can nevertheless be effectively used to calculate bulk properties with relative ease (as permitted by Bloch’s theorem). In 2D COFs and MOFs, one common use of *ab initio* methods is to characterize the layer spacing and stacking motifs, which can have a significant effect on material performance. Experimental techniques, such as XRD and SAED, can provide information about interlayer spacing in moderately ordered materials, but are unable to assign stacking configuration(s). As a result, it has become standard practice to include modeled patterns alongside experimental ones to aid with materials characterization. Sajid recently published a thorough review of computational methods that have proven successful in predicting the complex layer interactions of 2D-COFs with a variety of application focuses (Sajid, 2024). In the realm of *ab initio* methods, dispersion corrected density functional theory (DFT) and self-consistent-charge density functional tight binding (SCC-DFTB) are the most notable at accurately minimizing the various stacking configurations.

The Born-Oppenheimer approximation also lays the foundation for molecular mechanics (MM) methods where only the motion of the nuclei is taken into consideration when calculating the ground-state energy and structure of a material. Combined with the use of empirically derived force-fields to describe the bonded and non-bonded interactions within a system, these methods, which include molecular dynamics (MD) and Monte Carlo (MC), are more widely applicable to larger systems than *ab initio* methods. However, their accuracy is directly tied to the accuracy of the force-field and while there are general force-fields that perform moderately well, the best performing force-fields are system-specific. Sajid highlights that where *ab initio* methods are primarily used for calculating electronic properties, geometry configurations, and small length/time scale molecular interactions, MM methods are useful in calculating dynamical properties influenced by temperature and pressure and investigating molecular interactions on larger length/time scales (Sajid, 2024).

The primary motivating factor in deciding to use *ab initio* or MM methods are the relevant length and time scales associated with the application in question. There is not one method currently able to predict across all length and time scales. Herein lies the acute challenge of modelling polymers. While COFs and MOFs have a range of interactions across multiple length scales, their periodic structures make it comparatively simpler to elucidate these interactions with more accurate methods than is possible with polymers. Some authors have achieved success, however, with semi-empirical methods like SCC-DFTB (Mesta et al., 2019; Gooneie et al., 2017). As the tight-binding model involves calculating the electronic wavefunctions as a linear combination of atomic orbitals, SCC-DFTB is able to complete electronic structure calculations at speeds much faster than traditionally achieved by DFT with a minimal loss in accuracy (Gahrouei et al., 2024). Mesta *et al.* demonstrated this by using a tight-binding model to predict the HOMO-LUMO gap of conjugated donor-acceptor polymers for organic solar cells (Mesta et al., 2019). However, as discussed by Gooneie *et al.* in their review of computational modelling methods for polymers, (Gooneie et al., 2017) polymers display phenomena on one scale that often affects the phenomena on other scales, underscoring the need for a method that can precisely predict behavior across length and time scales. To date, the primary method for tackling this challenge is to combine methods into a multi-scale approach. This review by Gooneie *et al.* is recommended for an extensive discussion of multi-scale modelling with respect to polymers.

The challenge posed by the length and time scale requirements in modeling polymers and, to a lesser extent, POFs, carries through to modelling hybrid polymer-POF materials. For most applications of polymer-POF hybrids, MD and MC are the most reliable approaches as larger length/time scales and variations in temperature/pressure can be accounted for while still incorporating accurate atomistic interactions, depending on the force-field employed. Despite this, the amount of computational work on polymer-POF hybrid materials is limited. The only review of computational methods employed in modeling Polymer-MOF MMMs was published by Erucar and Keskin (2016). Though thorough, their review primarily focuses on the different gas permeation models that can be used to characterize membrane performance, which rely on results from MM simulations. One significant contribution to computational modelling of polymer-MOF MMMs was the work of Semino and co-workers, who published the first computational workflow that could probe the microscopic interactions of a polymer-MOF interface (Semino et al., 2016). The authors focused on the interactions of the polymer, PIM-1, with the zeolitic imidazolate framework, ZIF-8. Their published methodology focuses on merging the polymer and MOF and consists of several molecular dynamics (MD) simulations that equilibrate the monomer chains with the MOF surface. In this work, the authors determined that the interactions between ZIF-8 and PIM-1 were primarily attributed to interacting sites within energetically favorable distances, the rigidity of the polymer, and the availability of voids within the MOF. Another publication from the Maurin group studied functionalized variations of PIM-1 integrated with the MOF, UiO-66(Zr), the results of which were validated by experimental high-resolution TEM images (Carja et al., 2021). Other research groups have cited Maurin’s methodology for integrating polymers and MOFs, such as Kong and Liu who deployed this methodology in their computational study on CO_2_/N_2_ permeation through a MMM made with the porous organic cage, CC3, and PIM-1 (Kong and Liu, 2019).

As mentioned in the beginning of this section, the entire community of materials science must contend with the vast possibility of molecular combinations for both polymers and POFs. Even if the individual design spaces were fully uncovered, the community would still need to investigate the compatibility and target application performance of the compound materials. As it would be impossible to synthetically test all possible polymer-POF combinations, the onus lies with the computational community to seek out the most promising hybrid materials. Rather than exhaustively testing all combinations and then analyzing their properties, the more efficient route is to determine the intrinsic microscopic material properties that relate to the macroscopic performance, known as qualitative structure-property relationships (QSPRs). Understanding QSPRs then paves the way for application focused materials-by-design. The necessity for multi-scale modelling is emphasized again with a greater importance for speed in order to advance our technologies in an energy-demanding world.

Across the landscape of science and engineering, the last 10 years have witnessed an exponential increase in the use of ML. It has been utilized with great success across the field of materials science to speed-up the identification of QSPRs and to develop highly accurate force-fields. Again, as this review is primarily focused on polymer-POF hybrid materials, an overview of ML for the individual materials is addressed below only as a preface for contrasting the applications for polymer-POF hybrid materials.

There are several robust reviews that highlight successful ML strategies applied to MOFs for a variety of applications (Chong et al., 2020; Shi et al., 2020; Shi et al., 2020; Park et al., 2024; Mukherjee and Colón, 2021) and while approaches for MOFs are largely applicable to COFs, no review of ML on COFs has been published at this time. Therefore, we will touch on a few recent and high-impact applications of ML on COFs. The three main uses of ML in the following papers are to predict performance metrics which would normally be intensively calculated from MMs, to unveil the QSPRs related to the target performance, and to identify the optimal synthetic reaction conditions to produce crystalline COFs. In the following paragraph three seminal COF papers are discussed for each of these applications. The work on ML derived force-fields is addressed in a separate paragraph.

De Vos et al. (2024) used ML to accelerate the calculation of adsorption isotherms from a COF database in order to screen and predict high-performing COFs for post-combustion carbon capture. The group utilized their previously developed database of hypothetical COFs, ReDD-COFFEE, (De Vos et al., 2023), which contains 268,687 COFs, both 2D and 3D, that have been optimized with *ab initio* derived force-fields. A diverse subset of this database containing only 15,000 COFs was selected to train the ML algorithm. Their adsorption uptakes of CO_2_ and N_2_ were calculated using Grand Canonical Monte Carlo (GCMC). Once trained and validated, the ML algorithm accelerated the prediction of adsorption properties for the other 253,000 structures. Thus, the entire database could be screened to isolate the highest performing materials. While the database contained mostly hypothetical structures, the work by De Vos et al. (2024) highlights the promise of COFs for carbon capture and recommends target characteristics for designing future materials, such as pore size and linkage types. Ning *et al.* utilized ML to uncover the QSPRs of COFs from the CURATED database, (Ongari et al., 2019) containing 656 2D and 3D COFs, for SF_6_/N_2_ separation (Ning et al. 2024). The authors calculated adsorption isotherms of all COFs using GCMC and then trained four different ML algorithms on structural, energetic, and chemical features of the materials. After conducting multivariate analysis and comparing the accuracy of four different ML models, the authors concluded that void fraction and infinite dilution heat are the most significant predictors of SF_6_/N_2_ selectivity and framework regenerability. The authors also investigated the molecular-level interactions of the four highest performing frameworks with GCMC and DFT calculations. Finally, Kumar *et al.* used ML to classify crystallinity of experimental COFs and to identify the QSPRs most related to high surface area, yield, and crystallinity (Kumar et al., 2021). Their dataset contained 60 samples comprised of five different COFs experimentally synthesized in 12 different green solvents. This publication is particularly noteworthy as it utilizes cutting-edge computational methods to bridge computational and experimental practices in the interest of developing greener chemistry techniques. While Kumar *et al.* acknowledge that their model is preliminary and requires more training data, they demonstrate the current power of their model to accurately predict surface area and crystallinity of TpPa-2 and TpTta, both structures outside of their training set, synthesized in two separate solvents.

All of the works discussed above and many of the publications discussed in the cited reviews focus on the use of ML to predict QSPRs. However, ML has also been a powerful tool for developing highly accurate and fast force-fields. Park et al. (2024) reviews the current state of machine learning potentials (MLPs) for MOFs. Wiser and Zojer come to similar conclusions in their review by thoroughly comparing the forms and accuracy of existing MLPs in predicting the dynamical properties of MOFs (Wieser and Zojer, 2024). As many MLPs are trained on *ab initio* data, the bottleneck in advancing their accuracy and parameterization is the availability of large quantities of *ab initio* training data. Although large open-access *ab initio* databases such as QMOF (Rosen et al., 2021) and OpenDAC (Sriram et al., 2024) exist, even more data is required to continue parameterizing these potentials. Additionally, many of the applications of MLPs are very system specific and are therefore not widely generalizable. As an example, Huang *et al.* developed a MLP on DFT data to accurately investigate the dynamics of eclipsed stacking of two 2D COFs^174^. Their work highlights the importance of dynamic simulations to produce experimentally accurate layer stacking in 2D materials, but as their training set only includes two COFs, it also supports the claims of the previous review papers (Park et al., 2024; Wieser and Zojer, 2024) that while promising, MLPs are currently very dependent on DFT data and are thus, currently, much slower and less generalizable than classical force-fields.

There are many significantly thorough reviews on the use of ML to advance polymer predictions (Audus and de Pablo, 2017; Rohit et al., 2021; Tao et al., 2021; Zhao et al., 2023; Nguyen et al., 2022). We acknowledge that many of these reviews focus on the combination of ML and atomistic simulations. For a robust review on coarse-grained ML techniques for polymers, the authors recommend Nguyen (Nguyen et al., 2022) To briefly summarize the conclusions of these reviews, the primary challenges to ML on polymers are 1) the availability of large datasets with high-fidelity training data and 2) determining meaningful and computationally inexpensive descriptors to train the ML algorithm on for a target application. One of the largest open-access polymer databases, PolyInfo, contains 13,000 homopolymers with structure and property information (Otsuka et al., 2011). However, not all of the properties are tabulated for the entire database due to missing experimental work. Tao *et al.* exhaustively tested a variety of ML models and descriptors in predicting the glass transition temperature of polymers gathered from the PolyInfo database (Tao et al., 2021). They note that the experimental glass transition temperature was only included for 6,923 of the structures. The other 5,690 structures that they investigated from the database required computationally calculating the glass transition temperature, which only adds to the time and amount of necessary calculations necessary to complete the work, which underscores the need for more thorough databases. The review by Audus and de Pablo acutely articulates the necessity for and challenges with designing a complete polymer database (Audus and de Pablo, 2017).

The other challenge for ML applications of polymers is to determine meaningful descriptors that relate accurately to the target application. Many of the reviews comment on the widespread use of simplified molecular-input line-entry system (SMILES) strings as they are a simple representation of the structure, typically the monomer, and can generate additional topological or conformational descriptors. However, SMILES are not able to generate 3D properties, meaning that geometric features such as surface area or volume cannot be generated from SMILES strings (Zhao et al., 2023). It is also noted that SMILES are limited to homopolymers, (Nguyen et al., 2022) and there are conflicting conclusions whether models trained on monomer data sets perform accurately (Tao et al., 2021; Zhao et al., 2023; Nguyen et al., 2022). That is not to say that SMILES strings as descriptors are without their merits as they have proven to be powerful and very simple descriptors in many cases (Zhao et al., 2023; Nguyen et al., 2022). The general recommendation, though, seems for the community to continue investigating meaningful descriptors of polymers for ML.

Just as the lack of consistent data challenges the development of predictive ML algorithms, it also poses a significant challenge for developing MLPs, if not a greater challenge as most MLPs are trained on *ab initio* data. However, some progress has been made to develop MLPs for specific problem sets. Hong *et al.* developed a MLP from *ab initio* simulations of different length chains of polytetrafluoroethylene (PTFE) (Hong et al., 2021). Two different MLPs were trained: one that was trained on calculations that included explicit consideration for van der Waals (vdW) forces through the incorporation of a secondary bonding term and another dataset that negated these vdW terms. The authors conclude that the explicit incorporation of vdW forces significantly improved the accuracy relative to experimental data on the prediction of PTFE density, melting temperature, coefficient of thermal expansion and Young’s modulus. Additionally, while the vdW MLP was trained on PTFE chains between 7 and 10 carbons, it was able to accurately predict the density of a bundle of 390 carbon-chain. This is similar to the results Mohanty *et al.* obtained with their *ab initio* trained MLP, which was trained on monomers, dimers, and trimers of ethylene glycol, but performed well when extrapolated to tetraethylene glycol (Mohanty et al., 2023). While the model accurately scales, the authors note that in order to be applicable to other polymer systems, the training set would need to include clusters relevant to those systems. Similar to the state of MLPs for POF applications, more *ab initio* data is needed to train generalizable MLPs for polymers.

The application of ML to hybrid polymer-POF materials is very much in its infancy with very few publications. In contrasting the results of ML for POFs and polymers, the primary difference seems to be the availability of large and reliable datasets. While there are many large and accurate databases for COFs and MOFs which include complete property values, this is lacking for polymers and seems to be the primary bottleneck in advancing ML applications on polymers. As such, many of the ML studies on polymer-POF hybrid materials tend to utilize these vast databases of POFs and limit their study to a handful of well-characterized polymers. Guan *et al.* tested the highest number of polymers with a random forest algorithm trained on literature data of 648 MMMs for CO_2_/CH_4_ separation using 36 MOFs and 41 polymers (Guan Q. et al., 2022). Using the conclusions from their model on the high-performing traits of MOF fillers, the authors synthesized four MOF-polymer MMMs with Cu-THQ and Cu-CAT-1 as the MOFs, and Pebax-2533 and PIM-1 as the polymers. Cu-CAT-1 was determined to be the better performing MOF and when blended with 20% weight PIM-1, the permeability performance surpassed the Robeson upper bound. Daglar and Keskin used fewer polymers in their study to determine QSPRs of MOF-polymer MMMs for the separation of He/H_2_, He/N_2_, He/CH_4_, H_2_/N_2_, H_2_/CH_4_, and N_2_/CH_4_ (Daglar and Keskin, 2022). Four separate ML algorithms were trained to predict the gas uptake and diffusivity from the 2019 CoRE MOF Database (Chung, et al., 2019). The ML predicted values were then used to calculate the gas permeability and selectivity of 31,494 MOF-polymer MMMs with six different polymers, which were validated against experimental data and Maxwell model calculations. Finally, while the high-throughput study published by Aydin *et al.* did not utilize ML, their work is included in this discussion as it elucidated QSPRs of MOF-polymer MMMs and contributes to the field of computational materials discovery as it pertains to polymer-POF hybrid materials (Aydin et al., 2023). Using GCMC, the authors studied 1,193 MOFs, 589 COFs, and 4 polymers on the separation of CO_2_/N_2_, CO_2_/CH_4_, H_2_/N_2_, H_2_/CH_4_, and H_2_/CO_2_. Although Guan *et al.* tested the most polymers out of this selection of publications, the total number of structures in their dataset was 1,476 while the other works tested structures into the tens of thousands while only including less than 10 polymers. Again, the bottleneck in hybrid polymer-POF ML seems to be the lack of exhaustive and complete data on polymers. At the time of this review, no publications have been made utilizing or developing MLPs for hybrid polymer-POF materials.

To summarize, while computational methods, both traditional molecular methods and ML, have significantly advanced the field of materials discovery and expanded our understanding of structure-performance relationships, future discoveries rely on the availability of high-fidelity data. Machine learning models are currently the most promising tool for exploring structure combinatorics, QSPRs, and for designing force-fields that are accurate across length and time scales. Training these models, though, requires large, diverse, and complete datasets which is primarily lacking for polymers and will hinder the progress of uncovering high-performing polymer-POF hybrid materials.

## 4 Applications

Polymer-POFs hybrid materials represent an important class of advanced materials with significant potential across a wide array of applications, driven by the unique synergistic properties that arise from their covalent combination. POFs are known for their well-defined porous structures, high surface areas, and tunable chemical and physical functionalities, making them highly adaptable for various applications. Polymers, on the other hand, can contribute flexibility, processability, and mechanical robustness, enabling hybrid materials to combine these complementary features. The integration of polymers and POFs enhances the functionality and properties of their resulting hybrid materials, positioning these materials at the forefront of material science innovation. These hybrid materials have been extensively explored for broad applications in different fields such as membrane separation, environmental remediation, energy storage, fuel cells, catalysis, gas storage and separation, and biomedicine. In the following discussion, we feature several remarkable cases reported recently, emphasizing the strategies employed and the potential applications of these interesting materials, as demonstrated in the current literature.

### 4.1 Membrane separation

#### 4.1.1 Gas separation

Mixed matrix membranes (MMMs) enhance gas separation by increasing porosity and promoting interactions between fillers and the polymer matrix, which is due to the presence of functionalizable organic moieties. The gas transport process in mixed matrix membranes (MMMs) adheres to the solution diffusion model, wherein selectivity is determined by permeability, diffusivity, and solubility factors. Choosing suitable porous materials is essential for industrial applications, intending to enhance selectivity based on either diffusivity or solubility.

The separation of H_2_ and CO_2_ is crucial in the formation of H_2_ production process, where CO_2_ is a significant by-product. The market for this separation was evaluated at $150 million in 2020 (Low et al., 2008). Research suggests that ZIFs, also known as zeolitic imidazolate frameworks, are exceptionally efficient for separating H_2_ and CO_2_ gases (Bux et al., 2009). This is mostly owing to their remarkable thermal and chemical durability, exceptional selectivity, and minuscule aperture sizes. Ordoñez *et al.* conducted a study where they added 60 wt% ZIF-8 to Matrimid 5,218, resulting in an improvement in H_2_/CO_2_ selectivity (Ordoñez et al., 2010). Additional research conducted by Yang and Sanchez-Lainez *et al.* revealed that the incorporation of ZIF-7, ZIF-8, ZIF-11, or ZIF-90 into PBI or Matrimid 5,218 matrices enhances both the permeability and selectivity of H_2_ (Yang and Chung, 2013). In addition, various Metal-Organic Frameworks (MOFs) such as NH_2_–MIL-53(Al) and UiO-66(Hf)–(OH)_2_ have demonstrated enhancements in both H_2_ permeability and selectivity.

Solubility-based selectivity is crucial for applications such as CO_2_/N_2_ capture, CO_2_/CH_4_ natural gas/biogas upgrading, and ethylene/ethane separations. Studies have been conducted on various MOFs, including ZIF-7, ZIF-8, ZIF-71, ZIF-90, MOF-5, Cu3BTC2, and MIL-53 (Al). ZIF-7, possessing the most diminutive aperture size, exhibits flexibility that enables the adsorption of bigger gas molecules, commonly referred to as the gate-opening effect (Perez et al., 2017).

M-MOF-74, renowned for its open metal sites, exhibits a unique affinity for alkenes and CO_2_, hence improving its ability to separate these substances. Research indicates that the presence of Co- and Ni-MOF-74 nanocrystals in polyimide (6FDA-DAM) enhances the capacity of ethylene to pass through and improves the selectivity of ethylene over ethane (Supplementary Figure S4). This is attributed to the improved distribution and stronger bonding between the nanocrystals and the polymer matrix. Both Mg- and Mn-MOF-74 tend to clump together, creating empty spaces between them that only enhance the permeability of ethylene (Bloch et al., 2012; Bachman et al., 2016).

UiO-66 MOFs are renowned for their exceptional stability. The process of functionalizing these MOFs improves the interaction between particles and polymers, resulting in enhanced gas separation performance. The CO2 permeability and CO_2_/N_2_ selectivity of UiO-66-based mixed matrix membranes (MMMs) have been greatly enhanced through post-synthetic Ti exchange and simple metalated-ligand exchange using Na ions (Hu et al., 2017; Sarker et al., 2018).

#### 4.1.2 Liquid separation

##### 4.1.2.1 Desalination and water treatment

Water scarcity is a worldwide problem that impacts more than 1 billion individuals, and it is expected to intensify because of population growth and urbanization. Membrane-based separations, including ultrafiltration, nanofiltration, reverse osmosis (RO), and forward osmosis (FO), provide effective methods for producing purified water and mitigating pollution. Porous materials that are now emerging are regarded as promising membranes for the next-generation. This is because they possess high porosity, chemical resistance, mechanical strength, and exceptional permeability/selectivity (Cheng et al., 2018).

The utilization of MOFs in the process of water separation encounters difficulties because of the restricted durability of metal-containing secondary building units (SBUs) when exposed to water. Group IV metals such as titanium (Ti), zirconium (Zr), and hafnium (Hf), along with ligands that have low pKa values such as BDC and NH2–BDC, increase the thermodynamic stability of MOFs in water. In 2015, Liu et al. (2015) successfully produced Zr-MOF (UiO-66) polycrystalline membranes on alumina hollow fibers using *in situ* solvothermal synthesis. These membranes demonstrated remarkable capability to prevent the passage of multivalent ions and maintained stability in saline solutions for 170 h. Wang *et al.* enhanced the ability of UiO-66(Zr)–(OH)_2_ membranes to reject NaCl by sealing any defects in the membranes through a process called post-synthetic defect repair.

Mixed matrix membranes (MMMs) are extensively researched because of their convenient processing and efficient separation capabilities. Zhang et al. (2014) synthesized ZIF-8/poly (sodium 4-styrene sulfonate) (PSS) mixed matrix membranes (MMMs), which demonstrated a water flux of 26.5 L per square meter per hour per bar and a rejection rate of 98.6% for methyl blue. By incorporating MOFs into the polyamide layer, thin-film nanocomposite (TFN) membranes are created. These membranes exhibit enhanced water flow while maintaining equal levels of salt rejection in reverse osmosis (RO) with ZIF-8 and forward osmosis (FO) with UiO-66 processes.

COFs show great potential for water treatment because of their exceptional porosity, well-structured channel arrangements, and excellent resistance to water damage. The COF–LZU1 membranes, which are connected by imine linkages, exhibit a water permeability of 76 L m^−2^ h^−1^ bar−1 and a rejection rate of over 90% for dyes larger than 1.2 nm. Wang et al. introduced amine-rich COFs into the polyamide layer, resulting in an almost twofold increase in water flux, while still achieving 80% rejection of Na_2_SO_4_ (Wang C. et al., 2017). Valentino et al. fabricated a thin-film composite membrane with an asymmetric structure. The active layer of the membrane was made of COF and was created using interfacial polymerization of terephthalaldehyde and tris(4-aminophenyl) benzene monomers. This resulted in an enhanced rejection of Rhodamine-WT (Valentino et al., 2017).

##### 4.1.2.2 Pervaporation

Pervaporation is a highly promising technology used in various industries such as biorefinery, petrochemical, and pharmaceutical industries. It is particularly useful for separating liquid mixtures, whether they are binary or multicomponent. This technology can be applied to various processes such as dehydrating organic solvents, removing organic substances from water-based solutions, separating mixtures of organic compounds, and facilitating reversible reactions. Pervaporation is a process where the different components of a liquid mixture selectively absorb and pass through a membrane, evaporate on the other side due to low pressure, and are then collected as permeate (Cheng et al., 2018).

A recent work investigated the combination of 2D nanoporous COF–TpHZ and poly (ether sulfone) (PES) to create hybrid membranes for dehydration. The COF nanosheets exhibit spontaneous migration to the surface, resulting in the formation of graded membranes that are highly enriched with COF on the surface. The hydrophilic membrane demonstrated a penetration flux of 2.48 kg m^−2^ h^−1^ and a separation factor of 1,430 for the dehydration of ethanol/water. These values are not superior to those of commercially available membranes (Yang et al., 2018).

#### 4.1.3 Separation capacity

The membrane generated from PSP exhibits a notable capacity for separating heavy metal pollutants. The filtration setup demonstrates that the PSP-derived membrane, containing 60% MOF, achieves an 80% retention rate in the initial cycle and has a maximum separation capacity of 8 mg/g, Supplementary Figure S5. The performance of this membrane surpasses that of other membranes, such as PBMA membranes, MOF/PBMA blend membranes (which utilize non-polymerizable MOF, UiO-66), and activated carbon/PBMA blend membranes. The unpolluted polymer membrane has limited filtration performance, with a separation capacity of less than 800 parts per billion (ppb). The retention and capacity of the MOF/PBMA blend membrane are reduced due to inadequate compatibility and the creation of nonselective voids. The membrane composed of a blend of activated carbon and PBMA exhibits a retention rate of 37% and a capacity of 4.3 mg/g. However, its performance is hindered by the absence of adequate binding sites inside the pores (Zhang et al., 2015a). The PSP-derived membrane addresses these limitations by integrating the porosity and channels of MOFs with the flexibility and processability of polymers. The polymer contains nanosized MOF particles that have functional groups capable of undergoing polymerization. These particles are evenly distributed and chemically bonded to the polymer, which prevents them from clumping together and forming nonselective empty spaces. The copolymerization of MOFs and monomers improves the bonding between particles and polymers, resulting in a uniform composition at the molecular scale. The PSP-derived membrane exhibits higher separation performance due to its structural and compositional benefits.

### 4.2 Applications related to the environment

#### 4.2.1 COFs for greenhouse gas mitigation

Increasing worries regarding the escalating levels of atmospheric CO_2_ and its role in climate change have spurred initiatives to improve CO_2_ capture. An approach is altering COFs by including ionic liquids or carboxylic acid functionality. This is accomplished by chemically combining the phenolic hydroxyl groups of COFs with either (2-bromoethyl) triethylammonium bromide or succinic anhydride. These alterations enhance the absorption of CO_2_ by reducing the size of the pore walls and boosting the attraction between the attached functions and CO_2_ molecules (Huang et al., 2015; Dong et al., 2016). Sulfur hexafluoride (SF_6_) is a highly potent greenhouse gas that has a significantly greater warming potential than carbon dioxide (CO_2_). COF with a trifluoromethyl group has been discovered to be a highly efficient adsorbent for SF_6_. Incorporating–CF_3_ groups decreases the pore size of the COF, hence augmenting its affinity for SF_6_ molecules (Liao et al., 2021). Two distinct COFs, one in two dimensions (2D) and the other in three dimensions (3D) were created by introducing cobalt metal ions through post-modification. The cobalt-metalated COFs were created as chemosensors specifically designed for the detection of carbon monoxide gas (Yang et al., 2023).

#### 4.2.2 COFs for radioactive waste remediation

Uranium, employed in the nuclear sector, presents substantial environmental hazards as a result of its radioactivity and toxicity. The invention of an amidoxime-functionalized COF with a high adsorption capacity of 436 mg g^−1^ has resulted from efforts to efficiently remove uranium from the environment. This compound, formed by the Knoevenagel condensation reaction between 2,4,6-tris(4-formylphenyl)-1,3,5-triazine and 2,2′-(1,4-phenylene)diacetonitrile, exhibits sp^2^ carbon conjugation and displays outstanding fluorescent characteristics. The amidoxime groups function as receptors for efficient UO_2_^2+^ sequestration, leading to a rapid reduction in fluorescence intensity and a detection threshold of 8.3 nm, significantly lower than the U.S. Environmental Protection Agency’s threshold of 130 nm (Cui et al., 2020).

Radioiodine, which is present in nuclear waste, is another hazardous material that necessitates the use of efficient removal techniques. An earlier synthesized positively charged COF has shown effective absorption of iodine as a result of electrostatic attractions between the COF and iodine molecules (Zhai et al., 2024)

During a separate investigation, a hydrazone-linked COF was transformed to become a hydrazine-linked COF when exposed to reducing circumstances using sodium borohydride (NaBH_4_). This hydrazine-linked COF is particularly effective for iodine uptake from both gas and solution phases due to the abundance of NH groups in its framework, which allows hydrogen bond formation with radioactive iodine molecules.

#### 4.2.3 COFs as versatile tools for water purification

Carboxylic acid pesticides, which are utilized in agricultural practices, provide environmental hazards because of their teratogenic and carcinogenic characteristics, as well as their significant solubility. A COF modified with amino groups, produced by the thiol-ene click reaction, effectively removes these pesticides from water by interacting with their negatively charged carboxylate head groups (Ji et al., 2019).

Heavy metals, such as mercury, are highly poisonous and necessitate effective extraction from water. Thiol-functionalized COFs synthesized using a thiol–ene click reaction using bis (2-mercaptoethyl) sulfide and dithiothreitol, have exhibited significant adsorption capabilities for Hg(II) ions, with adsorption capacities of 586.3 and 535.5 mg g‒1. In addition, a sensor based on COF (covalent organic framework) was created to detect Cu(II) in seawater. The sensor’s conductivity was improved by depositing Au (gold) nanoflowers by electrochemical means (Wang et al., 2023).

Superhydrophobic COFs demonstrate high efficiency in separating oil and water. Imine-based COFs, which have been treated with perfluorooctyltriethoxysilane or employed in their non-fluorinated forms, demonstrate a remarkable oil/water separation efficiency above 99.5%. In addition, carboxyl-containing COFs that have been post-metalized with Ca_2_+ and Ni_2_+ demonstrate enhanced adsorption capabilities for organic dyes such as Congo red. This is achieved by exploiting electrostatic interactions in conjunction with hydrogen bonding and π-π stacking (Metal ion-assisted carboxyl-containing covalent organic frameworks for the efficient removal of Congo red - Dalton Transactions (Yue et al., 2019).

Amidoxime-functionalized COFs can be employed to identify and capture Roxarsone, a harmful organoarsenic chemical found in chicken feed, from wastewater. These covalent organic frameworks (COFs) can create robust hydrogen bonds with roxarsone, which enhances their effectiveness as adsorbents and fluorescence sensors (Chen et al., 2021).

### 4.3 Energy storage

#### 4.3.1 COFs in energy storage

The efficient storage of surplus energy from renewable sources is a crucial challenge that modern society must address by developing appropriate technology. Porous organic materials, particularly COFs, have great potential for use in charge-storage devices. This is because they have extensive pore systems, well-defined structures with long-range order, and the ability to accurately include a large number of redox-active functionalities (Beuerle and Gole, 2018).

DeBlase et al. showed that they successfully added anthraquinone units to the β-ketamine-linked 2D DAAQ-TFP-COF. When these modified electrodes were employed, the capacitance increased by 40.9 F g⁻^1^ (DeBlase et al., 2013). Furthermore, the capacitance did not significantly decrease even after more than 5,000 charge/discharge cycles. Applying COF thin films onto Au working electrodes resulted in a fourfold enhancement in capacitance compared to electrodes coated with DAAQ-TFP-COF powders (DeBlase et al., 2015). The process of electropolymerization of 3,4-ethylenedioxythiophene within the pores of DAAQ-TFP-COF thin films led to a significant tenfold enhancement in the current responsiveness. Furthermore, the capacitance was sustained even after undergoing more than 10,000 cycles (Mulzer et al., 2016). Xu et al. (2015) employed a post-synthetic modification strategy to synthesize [TEMPO]x-NiP-COFs with a maximum capacitance of 150 F g⁻^1^. The compound TpPa-(OH)_2_ exhibited specific capacitances of up to 416 F g⁻^1^, which is the highest recorded value for COFs up to now (Chandra et al., 2016).

#### 4.3.2 Lithium-sulfur batteries

COFs are being utilized as sulfur hosts in lithium-sulfur (Li-S) batteries to meet the increasing need for renewable energy. Within these batteries, the Li + ions undergo a reaction with sulfur to produce lithium polysulfides (LiPSs), which can migrate and result in the passivation of the electrode. Restraining the movement of LiPSs through lithiophilic interactions aids in immobilizing them and enhancing battery performance. A quinoline-linked triazine-based COF, created by reversible imine linkage through aza-Diels–Alder cycloaddition, showed promising electrochemical properties as a result of the interaction between nitrogen atoms in the triazine and quinoline components and lithium polysulfides (LiPSs). In addition, the expanded π-conjugated structure of the COF aided in the transfer of electrons during the conversion processes of Li-S (Liang Y. et al., 2022).

#### 4.3.3 Hydrogen storage

There are ongoing efforts to substitute carbon-based fossil fuels with clean fuels such as hydrogen gas to decrease greenhouse gas emissions in the transportation sector. Nevertheless, the extensive utilization of hydrogen fuel is constrained by the absence of cost-effective and secure storage methods. Creating adsorption-based COFs for hydrogen storage offers a promising alternative. Post-treatment methods such as impregnation, Li-doping, and functionalization can improve the ability of COFs to adsorb H_2_. A study showed that the hydrogen adsorption capacity was greatly enhanced by combining C60 impregnation and Li-doping. The process of impregnating C60 increases the surface area of the framework, hence enhancing the efficiency of Li-doping. The Li-doped sites subsequently engage with H_2_ molecules, augmenting their adhesion and retention inside the COF framework (Ke et al., 2017).

### 4.4 Proton-exchange membrane fuel cells

Functionalized COFs are employed as proton-conductive substances in proton-exchange membrane fuel cells. In these cells, hydrogen gas is oxidized to protons at the anode, causing proton diffusion through the conductive membrane to the cathode. At the cathode, protons attract electrons to generate electricity. This technique produces solely water as a secondary product, rendering it an environmentally friendly approach to energy generation.

To create COFs with excellent proton conductivity, sulfonic acid groups were added to the walls of the COFs using post-functionalization in a research study. In addition, an ionic COF demonstrated exceptional proton (H^+^) conduction due to the hydrophilic nature of COF decorated with Li + or Na + ions. This decoration enhances the density of hydrogen bonds between adsorbed water (H_2_O) molecules and mobile protons (H^+^), hence increasing the movement of protons. H_2_O adsorption was evaluated using density functional theory-based molecular dynamics. In addition, a β-ketamine type covalent organic framework (COF) was chemically modified with CuCl_2_ to produce a unique and highly proton-conductive COF for use in fuel cells. The carbonyl and amine functional groups present in the COF structure serve as chelating sites, allowing for the incorporation of copper (II) ions into the framework (Kang et al., 2020).

### 4.5 Catalyst

Industries favor heterogeneous catalysts over homogeneous ones due to their recyclability. Three palladium-anchored COFs were developed as heterogeneous catalysts for the Suzuki-Miyaura cross-coupling process, leading to increased production of bi-aryl compounds. The total rate of coupling product production was controlled by the coordination strength of bidentate ligands with N–N- and N–O-binding sites. Among these ligands, the COF with N–O-binding sites showed the fastest kinetics and highest coordination strength (Krishnaraj et al., 2022).In addition, a very effective heterogeneous catalyst for the asymmetric Henry reaction was created by attaching a chiral auxiliary to a pyridine-based COF. This resulted in significant improvements in both yield and stereoselectivity. In addition, a photocatalyst with different components was created by combining a COF that has bipyridine binding sites with iridium and nickel. This catalyst allows for C-N coupling reactions to occur with different types of substances. Finally, a catalyst with both acidic and nucleophilic catalytic sites was created by modifying a COF after its initial formation. This catalyst facilitated the cycloaddition reaction between CO_2_ and epichlorohydrin to produce cyclic carbonate. COFs that contained Br atoms showed higher catalytic activity because they had increased nucleophilicity.

Chang, Yaghi, and their colleagues created cobalt porphyrin COFs (COF-366-Co) that serve as catalysts for the electrochemical conversion of CO_2_ into other substances (Lin et al., 2015). The COFs exhibited significant activity and selectivity in inhibiting H_2_ production, with a turnover number (TON) of 34,000 within 24 h. This represents a four-fold increase in activity per electroactive Co compared to a molecular Co porphyrin counterpart, as well as a 10% enhancement in CO selectivity. Thin films that were oriented showed longer lives because they were less likely to separate from the electrode. This indicates that adjusting the thickness of the film could help achieve a balance between mass transfer and conductivity.

Cui and colleagues prepared a COF by synthesizing an imine-linked structure derived from tartaric acid. They then modified this COF with Ti(OiPr)_4_, resulting in a catalyst named CCOF-1 (Wang et al., 2016). This catalyst demonstrated high efficiency in catalyzing the asymmetric addition of diethylzinc to aldehydes. Specifically, it achieved a conversion rate of over 94% and an enantiomeric excess (ee) of over 90% for aldehydes containing electron-withdrawing groups. The catalyst was effectively retrieved and reused without any decrease in its activity or selectivity. Moreover, the (S)-pyrrolidine functionalized pores of imine-linked COFs exhibited reliable performance in catalyzing Michael addition processes, as observed in the TAPB-based COF.

COF-based catalysis offers the benefit of combining precise structures with the ability to be reused and integrated into continuous processes, which are characteristic of heterogeneous catalysts. However, frequently documented catalyst recycling experiments typically achieve complete conversion of the substrate but yield limited information (Jones, 2010). To assess COF-based catalysts more thoroughly, it is recommended to provide measurements of turnover numbers (TONs) and turnover frequencies (TOFs) for newly developed catalysts.

### 4.6 Gas storage and separation

#### 4.6.1 Clean energy gas storage

HOFs show great potential for storing environmentally friendly energy gases such as H_2_ and CH_4_. This is because they are composed of light elements, which allows for a large capacity to store gases based on weight. A preliminary instance, SOF-1, exhibited a moderate absorption of methane, measuring 106 cm³ g⁻^1^ at a pressure of 10 bar and a temperature of 195 K (Yang et al., 2010). Highly porous HOFs have demonstrated superior storage capabilities. For example, TTBI, which has a BET surface area of 2,796 m^2^ g⁻^1^, exhibited a notable H2 uptake of 243 cm³ g⁻^1^ (2.2 wt%) at 1 bar and 77 K (Mastalerz and Oppel, 2012). On the other hand, T2-g, which has the highest BET surface area among HOFs, displayed a methane uptake capacity of 47.4 mol kg⁻^1^ (437.4 v/v) at 115 K (Pulido et al., 2017).

#### 4.6.2 Adsorptive separation

The use of Hydrogen-bonded organic Frameworks (HOFs) for adsorptive separation is advocated as an energy-efficient method for chemical separation processes. An example of this is HOF-1, which showed effective separation of C_2_H_2_ and C_2_H_4_ with a high C_2_H_2_ uptake of 63.2 cm³ g⁻^1^ and a low C_2_H_4_ uptake of 8.3 cm³ g⁻^1^ at a temperature of 273 K and pressure of 1 bar (He et al., 2011). This resulted in a Henry separation selectivity of 19.3. Another very porous metal-organic framework, known as [Cu_2_(ade)_4_(H_2_O) _2_ (SiF6) _2_] (HOF-21), has a pore size of 3.6 Å and has fluorite binding sites that are easily accessible. This framework had an impressive capacity to adsorb acetylene (C_2_H_2_) at a rate of 1.98 mmol per gram and exhibited a selectivity for acetylene over ethylene of 7.1 (Bao et al., 2018). HOF materials often have weak binding sites, causing gas molecules with strong dipole or quadrupole moments to be less effectively adsorbed. This can lead to the opposite of the desired separations. As an illustration, HOF-76a had a significant ability to absorb C_2_H_6_ of 2.95 mmol g⁻^1^, while having a lower capacity for C_2_H_4_. The selectivity of C_2_H_6_ over C_2_H_4_ was 2.0. ZJU-HOF-1 also exhibited comparable separation capabilities (Zhang et al., 2021). A remarkable advancement in the separation of C_2_H_4_/C_2_H_6_ was accomplished using the flexible and durable microporous HOF-FJU-1, which is made from tetracyano bicarbazole (Yang Y. et al., 2021). This HOF, including pore sizes ranging from 3.4 to 3.8 Å, effectively facilitated the adsorption of C_2_H_4_ while preventing the adsorption of C_2_H_6_. As a result, it achieved an impressive C_2_H_4_ purity of 99.1%, establishing it as one of the most exceptional porous materials for this separation process. HOF-16, which contains unbound carboxyl groups, exhibited a significant disparity in the absorption of C_2_H_6_ and C_3_H_8_ molecules, with a difference of 76%. Additionally, it demonstrated a selectivity of 5.4 for propylene/propane separation under normal conditions. The HOF-30 with dia topology has been utilized for propylene purification as well (Yu et al., 2021).

Gas separation, namely the separation of CH_4_ and C_2_H_2_, is crucial in a wide range of industries. A quinoline-linked COF was produced by performing an aza-Diels–Alder reaction between an imine-linked COF and an enamide compound that includes a CF_3_ group. The reaction was facilitated by the presence of FeCl_3_ as a catalyst. This modified COF has demonstrated significant potential for separating CH_4_ and C_2_H_2_ as a result of the interactions between the hydrogen atoms in the gases and the fluorine or nitrogen atoms in the COF structure, which result in a higher attraction towards C_2_H_2_ (Wang et al., 2022). It is crucial to efficiently remove CO_2_ from natural gas and biogas. A COF-based mixed matrix membrane (MMMs) was fabricated using a polyethylene glycol monomethyl ether modified COF as a filler to improve the separation of CO_2_ gas (Liu Y. et al., 2021). In addition, a modified MOF-based mixed matrix membrane (MMM) was created by treating it with an imidazolium-based ionic liquid, [bmim][Tf_2_N]. This treatment improved the membrane’s ability to separate CO_2_ from CH_4_ effectively. The presence of anchoring ionic liquid increased the solubility of CO_2_ and decreased the pore size of the post-modified COF, hence restricting the diffusion of CH_4_. In addition, a COF membrane was utilized for the separation of ethylene and ethane, with the membrane being coated with a layer of ionic liquid that contains silver ions. At first, the membrane had a channel size that was bigger than the molecules, which led to a low level of selectivity. Following treatment with the ionic liquid, the size of the channel was decreased, resulting in an improved selectivity for ethylene over ethane. This was achieved through the creation of bonds between ethylene molecules and silver ions, which further reduced the channel size and prevented the transportation of ethane (Liang X. et al., 2022).

### 4.7 Biomedical applications

#### 4.7.1 Drug delivery

Polymer-MOF hybrids present innovative prospects in the realm of drug delivery, leveraging the unique properties of MOFs such as high surface area and porosity, alongside the versatility of polymers. These hybrids have been explored for their potential to enhance the controlled release of therapeutic agents, ensuring targeted and sustained delivery. The integration of biocompatible polymers with MOFs can lead to systems that not only protect the active pharmaceutical ingredients but also offer a responsive release triggered by specific stimuli, thus opening new avenues for advanced treatment strategies. Supplementary Figure S6 provides an overview of these larger, solid composites where MOFs are integrated into polymer matrices to enhance properties like catalytic activity or porosity (Schmidt, 2020). Thin films or layers where MOFs and polymers are combined, often aim to improve gas separation or filtration capabilities.

In Hong and Xu’s group, they pioneered the development of covalently connected MOFs and polyethylene glycol (PEG) hybrids, aiming to enhance imaging and drug delivery capabilities (Lee et al., 2023). Inspired by the positive effects of PEGs, they explored covalent connections between MOFs and PEGs using copper-catalyzed azide-alkyne cycloaddition (CuAAC). Specifically, they prepared azide-functionalized UiO-66-N3 MOFs and loaded the anticancer drug doxorubicin (DOX) into these MOFs. Alkyne-terminated PEGs were then covalently bonded to the MOFs via triazole formation, resulting in DOX/UiO-66-PEG conjugates labeled with the radioisotope 89Zr (DOX/89Zr-UiO-66-PEG). Spectroscopic techniques confirmed the successful triazole formation within this composite. To further enhance tumor targeting, they introduced a thiol-terminated F3 peptide (HS-Cys-F3) into the DOX/UiO-66-PEG conjugates via Michael addition. Cellular uptake studies demonstrated significantly higher uptake of DOX/UiO-66-PEG-F3 compared to simple DOX/UiO-66-PEG, and *in vivo* results revealed excellent targeting behavior of UiO-66-PEG-F3 (Lee et al., 2023).

In the realm of oncological pharmacotherapy, the advent of diamondoid supramolecular organic frameworks (dSOFs) has marked a significant leap forward in drug delivery systems (Supplementary Figure S7) (Li et al., 2022) These frameworks are adept at encapsulating anionic drugs, such as pemetrexed (PMX), used in the treatment of non-small-cell lung cancer and malignant mesothelioma. The encapsulation process is facilitated by the dSOFs’ ability to form cooperative ion-pair electrostatic interactions within their porous structure, as evidenced by dynamic light scattering (DLS) and zeta potential analyses. This innovative approach not only simplifies the drug loading process but also enhances the intracellular delivery of therapeutics via the enhanced permeability and retention (EPR) effect, characteristic of nanoscale macromolecular carriers. Consequently, dSOFs stand out as a promising *in situ* carrier system, potentially revolutionizing the delivery of negatively charged antitumor drugs and mitigating the complexities and costs associated with traditional liposomal and albumin-drug conjugates (Li et al., 2022).

In the pursuit of safe and effective cancer therapy, the development of agents capable of accurately differentiating tumors from normal healthy tissues is of utmost importance. Active targeting has emerged as an effective technique for tumor recognition, leveraging specific interactions with tumor-associated receptors (Yu et al., 2023).

In this work, the team introduces a folate-functionalized nanoscale covalent organic framework (FATD nCOF) designed for highly specific cancer cell targeting. The FATD nCOF is synthesized through a straightforward post-synthetic modification of the COF surface, resulting in excellent water dispersibility and a high loading capacity for various anticancer drugs. Importantly, the biocompatible FATD nCOF selectively internalizes within cancer cells enriched with folate receptors (FRs), thereby enhancing the therapeutic efficacy of the loaded drug, Withaferin A (Wi-A). Comparative analyses of FR-positive and FR-negative tumor xenografts reveal the enhanced selective antitumor activity of FATD@Wi-A nanotherapeutics. These findings underscore the promise of FATD nCOF as an active targeting agent for *in vivo* tumor recognition, opening new avenues for the development of state-of-the-art COF-based nanomedicines for future therapeutic applications (Rejali et al., 2023; Yu et al., 2023).

#### 4.7.2 Biologics chromatography

In the recent study published in the Journal of Chromatography A, researchers have innovatively employed a post-modification approach to enhance the separation capabilities of covalent organic frameworks (COFs) for isomer analysis (Ma et al., 2022; Rejali et al., 2023). The newly developed TzDHNDA-PEAA COF, has been synthesized through an esterification reaction, which integrates [(1-phenylethyl)amino]acetic acid (PEAA) into the TzDHNDA framework. This modification introduces benzene rings, secondary amines, and carbonyl groups, which significantly bolster the π-π interactions and hydrogen-bonding forces within the COF structure (Yue et al., 2019).

Successful coating of TzDHNDA-PEAA onto capillary columns, which are pivotal for gas chromatographic applications. The TzDHNDA-PEAA coated columns demonstrate superior performance in the baseline separation of isomers, achieving high resolution and precision. Notably, these columns require lower temperatures for fluoroaniline separation and shorter times for chloroaniline separation compared to commercial columns such as InertCap WAX, InertCap 5, and InertCap 17,013. The enhanced separation efficiency is attributed to the synergistic effect of the introduced functional groups, which improve the interaction between the stationary phase and the isomers (Ma et al., 2022).

Zhang and co-workers performed post-modification strategies in fine-tuning the properties of COFs in which the novel COFs were tailored for the high-resolution separation of isomers (Zhang et al., 2024). In this study, novel microspheres were developed by constructing MOFs covalently linked with COFs on silica to serve as stationary phases for HPLC. The resulting SiO_2_@NH_2_-UiO-66@CTF column exhibited remarkable properties. Given the highly rigid π-conjugation system of the COF TzDHNDA, the column demonstrated superior hydrophobicity and aromatic selectivity. Notably, it achieved an exceptionally high molecular shape selectivity with an α value of 6.01. Second, the presence of Zr^4+^ sites and hydrogen bonding sites in NH2-UiO-66 contributed to its promise in separating heteroatomic contaminants. Third, the column exhibited a degree of hydrophilicity due to oxygen-containing groups. These multifunctional properties collectively enabled the successful separation of organic phosphorus pesticides from complex food matrices using the SiO_2_@NH_2_-UiO-66@CTF column. The approach highlights the potential of MOFs@COFs hybrid materials as efficient SPs for HPLC applications, providing a versatile platform for complex sample separation.

## 5 Conclusion and perspective

In conclusion, the exploration of polymer-porous organic frameworks (polymer-POFs) hybrid materials illustrates a promising avenue in materials science, particularly in addressing the inherent limitations of traditional POFs. By integrating polymers into POFs, namely combining the flexibility and processability of polymers with the robustness and tunable porosity of POFs, researchers have successfully enhanced the mechanical properties, chemical stability and versatility, and overall performance of these resulting hybrid materials. These developments suggest that polymer-POFs may offer enhanced functionality and versatility compared to conventional materials in a range of applications. The ability to precisely control pore environments and surface features is enabled by the covalent integration of polymers and POFs, which endows these hybrid materials with high flexibility for specific applications, including gas separation, catalysis, and biomedical engineering. Moreover, the capability of manipulating the physical and chemical properties of these resulting hybrid materials at the molecular level enables substantial development in a wide variety of applications. The broad array of synthetic strategies, including polymerization within, on, and among POFs, showcases the versatility and prospective impact of these hybrid materials to revolutionize fields like membrane technology, environmental remediation, and energy storage. The advancement of these hybrid materials not only leverages the advantages of both polymers and POFs, but also opens new pathways for the preparation of advanced materials with unprecedented functionalities and efficiencies.

Looking forward, the field of polymer-POF hybrid materials is poised for significant growth, with numerous research avenues to be explored, in order to fully unlock their potential. First of all, further investigation is required to ascertain the scalability of polymer-POF hybrid materials for industrial applications. Even though the current research has demonstrated the possibility of synthesizing these materials on a laboratory scale, the translation of these methods in the lab to industrial-scale synthesis will require addressing several challenges such as cost, reproducibility, and the development of environmentally sustainable synthesis processes. The integration of green chemistry principles into the synthesis of polymer-POF hybrid materials could play a crucial role in making these materials more attractive for commercial applications.

One of the most promising directions lies in the development of stimuli-responsive materials, enabling the design and preparation of smart materials that are able to adapt to changing environmental conditions. By leveraging the dynamic nature of polymers and the structural tunability of POFs, researchers can design materials that respond to environmental changes such as temperature, pH, or light. These materials could have transformative impacts on fields such as smart drug delivery, where the release of therapeutic agents could be precisely controlled in response to specific biological conditions.

Additionally, further exploration into the synergistic effects between different polymers and POFs could lead to the discovery of new properties and applications. For instance, the development of multi-functional hybrid materials that can simultaneously perform gas separation and catalytic functions could significantly impact energy and environmental sectors.

Another critical area for future research is the integration of polymer-POF hybrid materials into electronic and optoelectronic devices. The ability to fine-tune the electronic properties of these materials through chemical modification of the polymer or POF components opens up possibilities for their use in sensors, light-emitting devices, and energy storage systems. In addition, the exploration of new types of POFs with unique electronic properties, such as conductive MOFs or COFs, in combination with conductive polymers, could lead to the development of highly efficient hybrid materials for electronic and energy-related applications.

Furthermore, the exploration of polymer-POF hybrid materials in the context of environmental sustainability presents a compelling research direction. These materials have already shown promise in areas such as CO_2_ capture and water purification. Expanding their application to other environmental challenges, such as the removal of emerging contaminants from water or the sequestration of hazardous waste, could provide significant societal benefits. As the field progresses, interdisciplinary collaboration between chemists, materials scientists, and environmental engineers will be essential to fully realize the potential of polymer-POF hybrids in addressing some of the most pressing environmental issues of our time.

Moreover, advancing the understanding of the interactions between polymers and POFs, either at the atomic/molecular level or at the material level, will provide deeper insights into the design of materials with tailored properties. Computational chemistry, along with machine learning (ML) and artificial intelligence, is a powerful tool to offer us a profound understanding for the design, synthesis, function, properties and applications of these polymer-POFs hybrid materials. Up to now, in the majority of the reported studies, the training of ML models has been conducted using a relatively small number of POF structures (mainly MOF and COF). This lies primarily in the difficulty of utilizing ML with polymers, which results from the limited number of meaningful descriptors and lack of consistent data. Conversely, there are several highly reliable COF and MOF databases that harvest consistent literature data for ML and high-throughput studies. To make greater advances in studying the compatibility of various polymers with organic frameworks via ML and high-throughput methods, specifically those which rely on computational data, further progress must be made on developing polymer descriptors and developing databases with reliable and abundant polymer data.

By focusing on these areas, the field of polymer-POFs hybrid materials stands at the cusp of revolutionizing material science and engineering, offering solutions to some of the pressing challenges in both chemistry and modern technology and industry.
