# Supplementary material for: Covalent integration of polymers and porous organic frameworks
Source: Front Chem. 2024 Dec 18;12:1502401. doi: 10.3389/fchem.2024.1502401 (PMC11688193; doi:10.3389/fchem.2024.1502401)
Supplement: Supplementary file 1 [file DataSheet1.pdf]

Covalent Integration of Polymers and Porous Organic Frameworks

Md Amjad Hossain[1], Kira Coe-Sessions[1], Joe Ault[1], Felix Gboyero[1], Michael J. Wenzel[1], Bhausaheb Dhokale[1], Alathea E. Davies[1], Qian Yang[2], Laura de Sousa Oliveira[1]*, Xuesong Li[1]*, and John O. Hoberg[1]*

[1] Department of Chemistry, University of Wyoming, Laramie, 82072 WY, USA

[2] Center for Advanced Scientific Instrumentation, University of Wyoming, Laramie, 82072 WY, USA

Supplementary Material

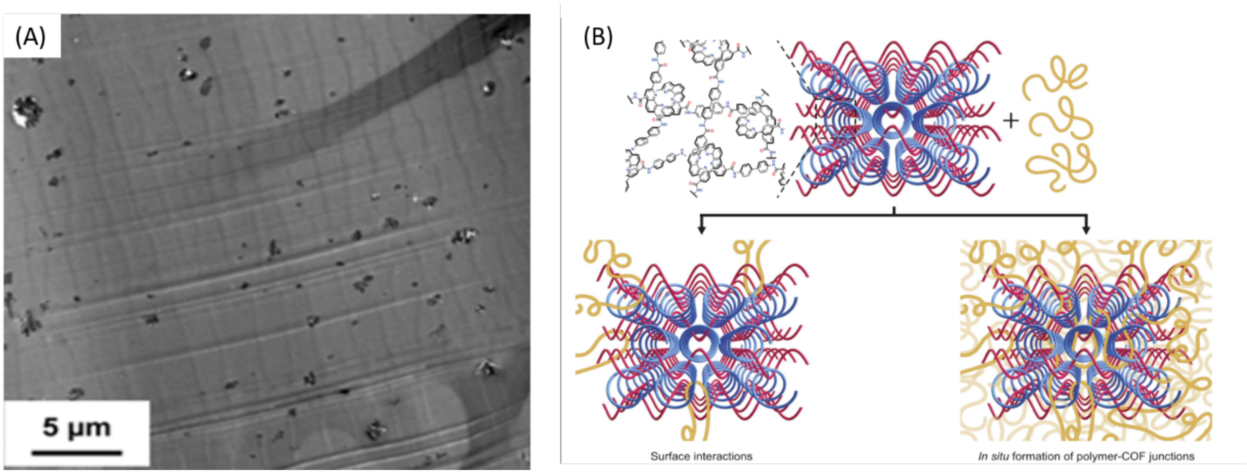

Figure S1 | (A). TEM image of PMMA-MW (3 wt %) showing well-dispersed MW nanocrystals in the PMMA matrix. (B) Polymer-COF interactions. Depending on the polymer, its matrix may either interface only with the surface of the woven COF particles or form so-called polymer-COF junctions. In these junctions, individual polymer chains penetrate the porous, 3D woven COF crystals and decorate the surface to interact with the polymer matrix. Reprinted Permission(Neumann et al., 2024b)

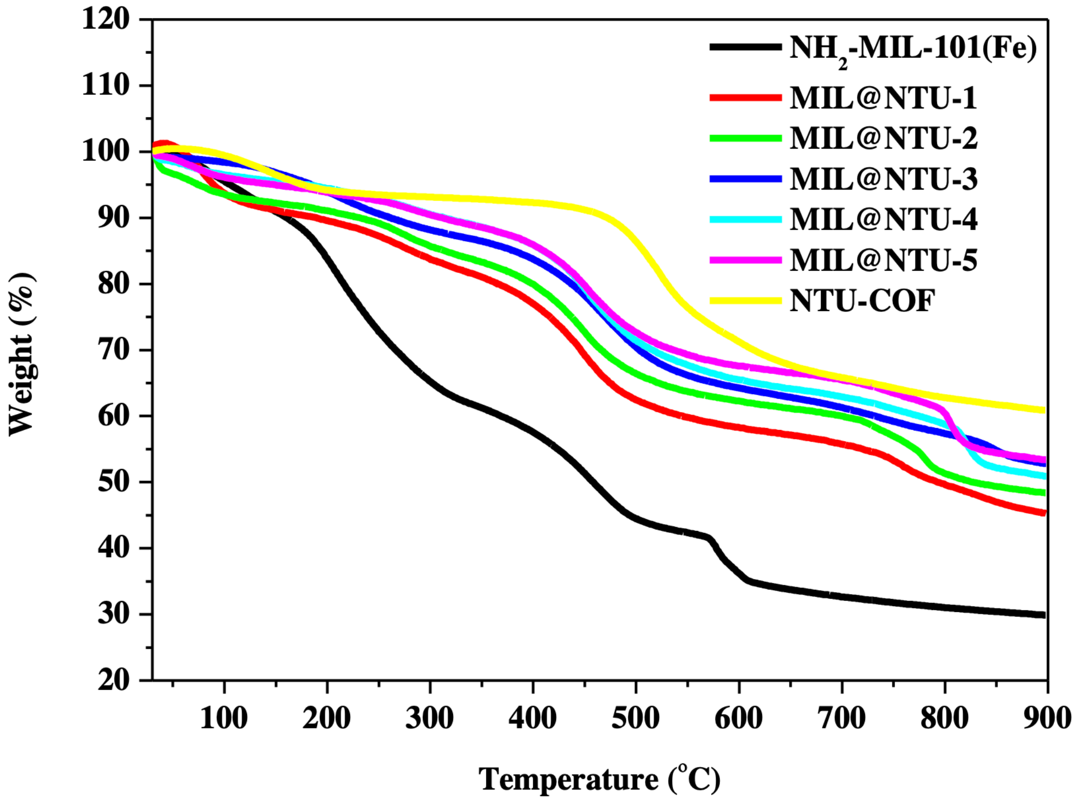

Figure S2 | TGA profiles of NH_2-MIL-101(Fe), MIL@NTU-1, MIL@NTU-2, MIL@NTU-3, MIL@NTU-4, MIL@NTU-5 and MIL@NTU. Reprinted permission(Cai et al., 2019b)

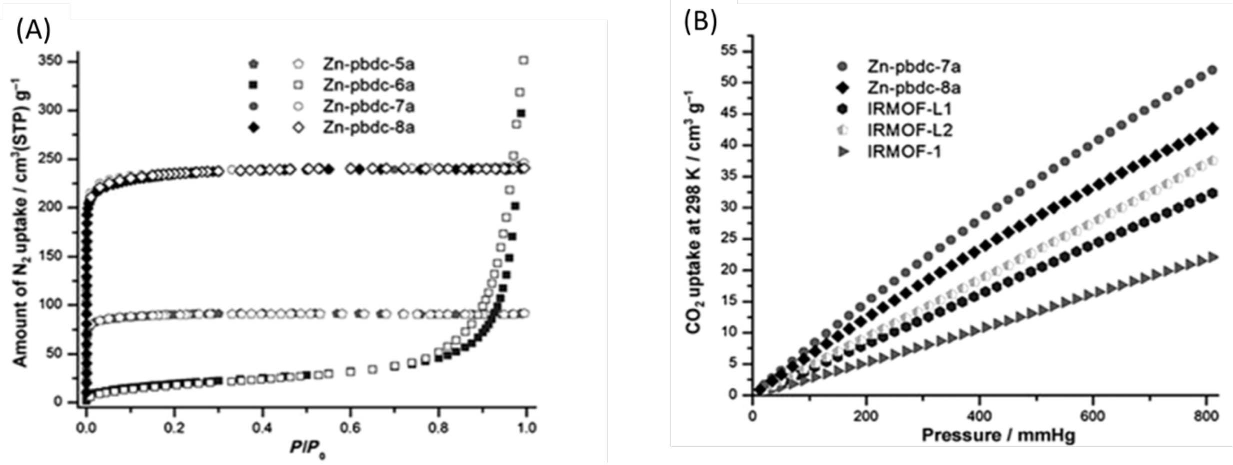

Figure S3 | (A) N_2 sorption isotherms for polyMOFs; (B) CO_2 adsorption isotherms at 298 K.(Furukawa et al., 2013; Zhang et al., 2015g; Bentz et al., 2020b). Reprinted permission(Bentz et al., 2020b).

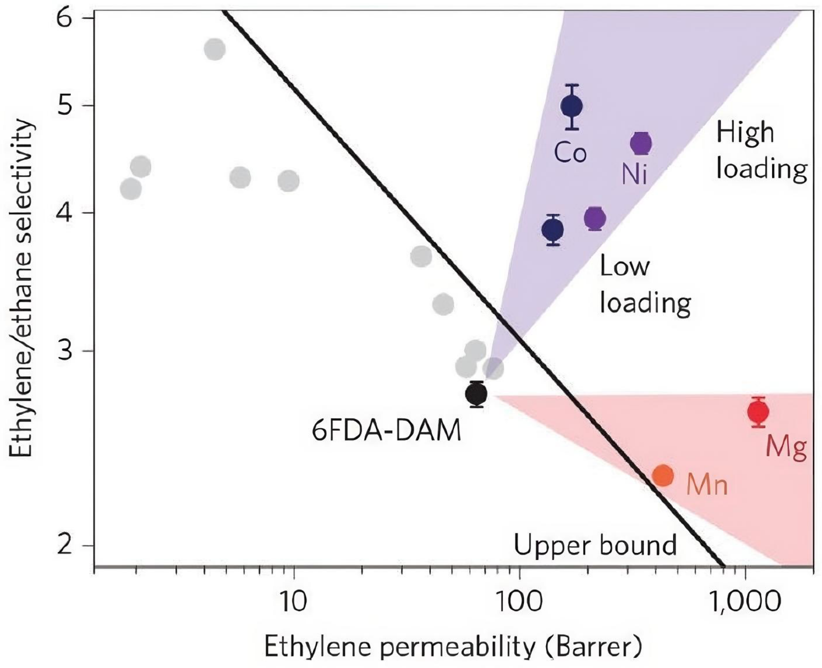

Figure S4 | Ethylene/ethane separation performance for MMMs containing M-MOF-74 (M refers to the metal building block). *(Cheng et al., 2018)*

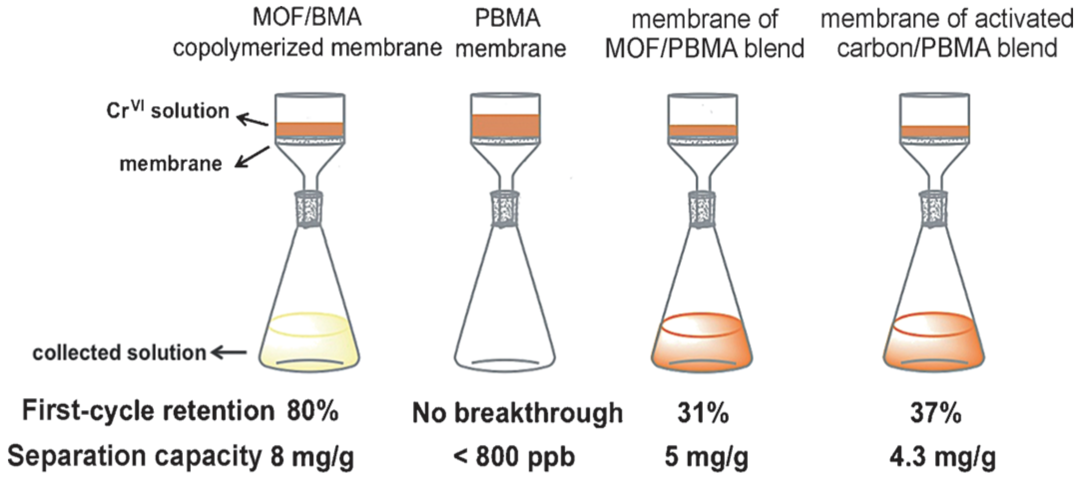

Figure S5 | Cr^{VI} ion separation capability of a MOF/BMA copolymer.(Zhang et al., 2015c)

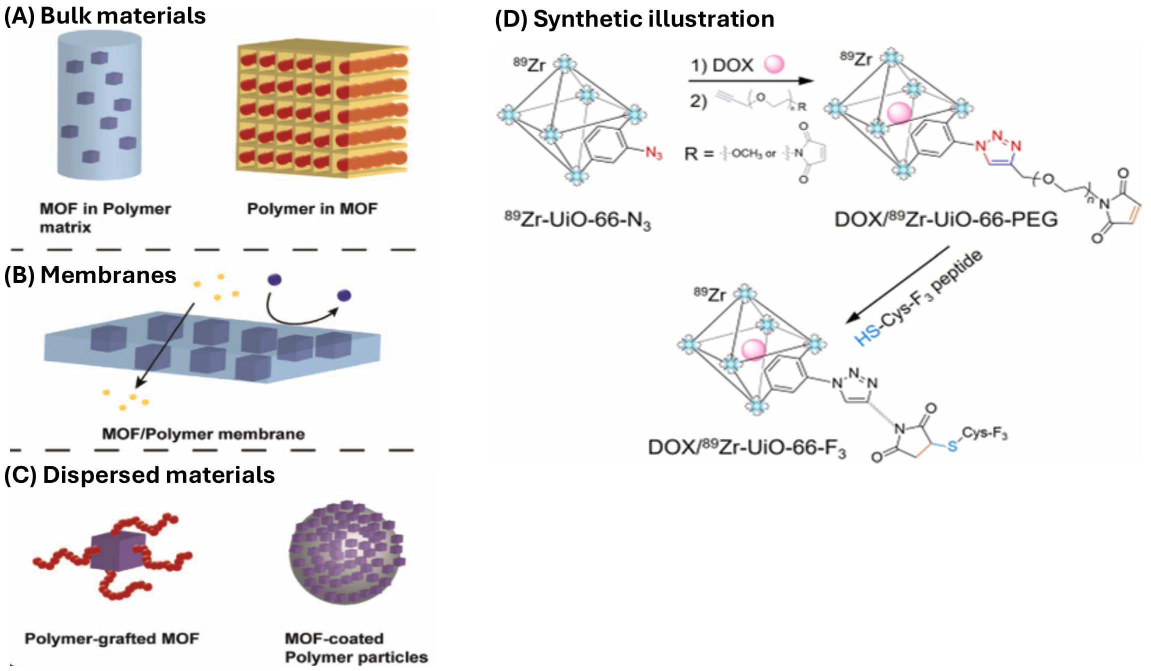

Figure S6 | Diverse configurations of hybrid materials combining metal-organic frameworks with polymers, showcasing (A) bulk composites, (C) dispersed matrices, and (B) membrane forms. Additionally, it includes a schematic illustration (D) of the step-by-step synthesis process for DOX/89Zr-UiO-66-F3_88, involving the combination of UiO-66-N3 with doxorubicin (DOX) and a specific peptide chain, resulting in the final compound.(Schmidt, 2020; Lee et al., 2023b)

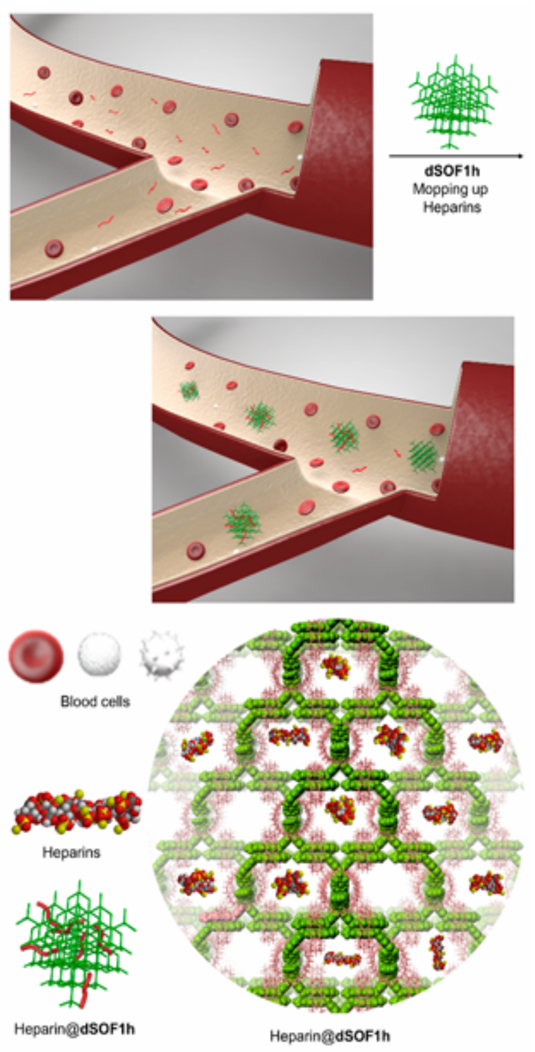

Figure S7 | Schematic illustration of the step-by-step synthesis process for dSOFh, which neutralizes the anticoagulant function of UFH and LMWHs. The diagram shows how dSOFh binds to heparins, preventing their anticoagulant effects while leaving blood cells unaffected.(Li et al., 2022)
